# Bidirectional Interplay Between Tumor Vaccines and the Tumor Microenvironment: Mechanisms, Cold-to-Hot Conversion, and Combination Strategies

**DOI:** 10.3390/vaccines14080658

**Published:** 2026-07-27

**Authors:** Zhangzhou Shen, Qinqin Feng, Fen Wang, Houqiang Luo

**Affiliations:** 1 Hubei Key Laboratory for Kidney Disease Pathogenesis and Intervention, Hubei Provincial Engineering Research Center of Immunotherapy Drugs for Renal Tumors, Medical School, Hubei Polytechnic University, Huangshi 435003, China; chenzhangzhou@hbpu.edu.cn; 2 College of Animal Sciences, Wenzhou Vocational College of Science and Technology, Wenzhou 325000, China; 3 Department of Obstetrics, Huangshi Maternity and Children’s Health Hospital, Affiliated Maternity and Children’s Health Hospital of Hubei Polytechnic University, Huangshi 435003, China; fqq20200710@163.com (Q.F.); wf20251020@163.com (F.W.)

**Keywords:** cancer vaccine, tumor microenvironment, cold-to-hot conversion, immune checkpoint inhibitor, neoantigen, combination immunotherapy, biomarker

## Abstract

Therapeutic cancer vaccines are designed to initiate tumor-specific immunity, yet their clinical success depends not only on antigen selection but also on the capacity to overcome the profoundly suppressive tumor microenvironment. Within tumors, abnormal vasculature, hypoxia, nutrient competition, acidic pH, and suppressive myeloid and stromal cells collectively constrain antigen presentation, T-cell priming, trafficking, and effector function, often converting otherwise immunogenic vaccination into an ineffective immune stimulus. Recent advances in neoantigen discovery, dendritic cell engineering, and nucleic acid-based vaccine platforms have improved the precision of antigen delivery, but these gains remain insufficient unless vaccine-induced responses can be sustained and executed within the hostile metabolic and immunologic landscape of the tumor niche. In this context, the tumor microenvironment is not merely a barrier to be overcome, but an active determinant of vaccine outcome that shapes immune editing, promotes exhaustion, and limits intratumoral expansion of cytotoxic lymphocytes. Accordingly, the most promising therapeutic strategies now combine vaccination with checkpoint blockade, radiotherapy, stromal remodeling, or metabolic reprogramming to recondition the tumor ecosystem and permit productive antitumor immunity. Here, we discuss how tumor microenvironmental constraints govern vaccine performance, review emerging platform technologies, and outline combinatorial strategies aimed at converting immune priming into durable tumor control.

## 1. Introduction

### 1.1. Therapeutic Cancer Vaccines: Concepts, Platforms, and Challenges

What they are. Therapeutic cancer vaccines are designed to stimulate the immune system to recognize and attack established tumors, rather than prevent cancer in the first place. They remain a rapidly evolving immunotherapy platform, with renewed momentum from neoantigen discovery, dendritic-cell approaches, and nucleic-acid vaccine technologies.

What they do. These vaccines target tumor-associated antigens (TAAs), tumor-specific antigens (TSAs), and personalized neoantigens to activate adaptive immunity through exogenous antigen presentation, enabling specific recognition and elimination of tumor cells [1,2,3,4]. In recent years, the maturation of whole-exome sequencing (WES), RNA-seq, and HLA-binding prediction algorithms has made it possible to screen immunogenic neoantigens even in tumors with low mutational burden [5,6,7,8,9,10,11].

Main platforms. Multiple vaccine platforms are currently under active development, each with distinct advantages and limitations. mRNA vaccines offer rapid production and strong immunogenicity but require lipid nanoparticle delivery systems [12,13,14,15,16,17,18]. Peptide vaccines are safe and easy to synthesize but often require potent adjuvants [19]. Dendritic cell vaccines can present multiple antigens but are costly and time-consuming to manufacture [20,21]. Viral-vectored vaccines possess intrinsic adjuvanticity but may be limited by pre-existing immunity [22,23,24,25]. DNA vaccines and universal mRNA platforms are also rapidly evolving [26,27,28,29].

Approved examples. In melanoma, mRNA-4157 combined with pembrolizumab significantly prolonged recurrence-free survival in adjuvant treatment [12,15]. The individualized mRNA vaccine autogene cevumeran, in a phase I trial (NCT03289962) combined with atezolizumab for solid tumors, induced multi-epitope CD4^+^/CD8^+^ T-cell responses lasting over 23 months, although three grade 4/5 serious adverse events occurred [16,17]. The heterologous prime-boost vaccine GRANITE (ChAd68 + samRNA) plus nivolumab and ipilimumab, in a phase I/II trial (NCT03639714), elicited neoantigen-specific CD8^+^ T-cell responses in 100% of evaluable patients (lasting >52 weeks) [12,18]. In a phase I trial of the long-peptide vaccine NEO-PV-01 combined with pembrolizumab and chemotherapy as first-line treatment for NSCLC, the objective response rate (ORR) among successfully vaccinated patients was 69%; however, 45% of enrolled patients could not receive the vaccine due to disease progression or manufacturing failure [12,19].

Why efficacy is limited. Despite these encouraging results, the overall response rate of single-agent vaccines remains low, particularly in patients with advanced disease, and long-term relapse control remains insufficient [3,30]. The primary reason lies in the immunosuppressive tumor microenvironment (TME), which creates physical, cellular, and metabolic barriers that block vaccine-induced immunity—a challenge that will be discussed in detail in Section 2.

Current directions. To overcome these limitations, current research is focusing on rational combination strategies—integrating vaccines with immune checkpoint inhibitors, TME modulators, chemoradiotherapy, oncolytic viruses, and advanced delivery systems—as well as biomarker-driven patient stratification and personalized neoantigen design. This review systematically examines these approaches and outlines future directions for the field.

### 1.2. The Tumor Microenvironment (TME) as a Key Barrier to Vaccine Efficacy

The TME is a dynamic and complex regulatory network composed of tumor parenchymal cells, infiltrating immune cells, stromal fibroblasts, extracellular matrix, and multiple soluble factors and metabolites [31,32,33,34]. Based on the abundance, activation status, and spatial distribution of immune cells, tumors are classified into immunologically “hot” (inflamed), “cold” (immune-desert), and immune-excluded phenotypes [35,36,37,38]. Most clinical solid tumors are of the immune-desert or immune-excluded types, which not only show a lack or spatial segregation of immune cells but also exhibit inflammatory dysregulation, metabolic reprogramming, and aberrant expression of immune-escape molecules, thereby hindering cancer vaccines at every step–antigen presentation, immune activation, cell infiltration, and effector killing [35,36].

### 1.3. The Immunosuppressive Tumor Microenvironment: A Multilayered Barrier to Vaccine Efficacy

The immunosuppressive environment in cancer is a coordinated, adaptive ecosystem that actively prevents effective antitumor immunity rather than simply reflecting passive immune failure. It is built from malignant cells, stromal cells, endothelial cells, fibroblasts, extracellular matrix, and infiltrating immune populations that together create a niche that excludes, exhausts, or reprograms effector lymphocytes.

Cellular architecture. At the center of the tumor microenvironment are cancer cells that shape immunity through antigen loss, altered cytokine secretion, immune checkpoint ligand expression, and metabolic rewiring [30,32,39]. Surrounding them, cancer-associated fibroblasts, abnormal vasculature, and dense extracellular matrix can physically restrict T-cell infiltration and create spatial barriers to immune cell access [32,39,40,41,42]. Myeloid populations, especially tumor-associated macrophages and myeloid-derived suppressor cells, are major suppressive drivers because they secrete inhibitory mediators and limit antigen presentation [43,44,45,46,47,48]. Regulatory T cells further reinforce tolerance by suppressing dendritic-cell function and effector T-cell activation [49,50,51].

Soluble suppressive signals. The tumor milieu is rich in inhibitory cytokines and growth factors, especially TGF-β, IL-10, VEGF, and related mediators that dampen T-cell priming and cytotoxic function [43,52]. These signals also promote immune exclusion, angiogenesis, matrix remodeling, and polarization of myeloid cells toward suppressive phenotypes [53,54,55,56]. At the same time, checkpoint pathways such as PD-1/PD-L1 and CTLA-4 impose brakes on T-cell activation and sustain functional exhaustion within the tumor [57]. Together, these pathways convert what should be an inflamed tissue response into a state of chronic immune paralysis.

Metabolic suppression. A defining feature of the immunosuppressive tumor microenvironment is metabolic competition. Tumors consume large amounts of glucose and amino acids, especially tryptophan, while generating lactate, carbon dioxide, and protons that acidify the local milieu. This deprives T cells of essential nutrients and impairs proliferation, cytokine production, and cytotoxicity [43,53,54,55,56]. Acidic pH, hypoxia, and low nutrient availability also favor suppressive myeloid cells and reduce the efficacy of dendritic-cell priming. Thus, the tumor microenvironment suppresses immunity not only through receptor-mediated inhibition but also by imposing a hostile metabolic landscape [57].

Immune exclusion and exhaustion. Many tumors are not immunologically “cold” because they lack immune cells entirely, but because infiltrating lymphocytes are rendered dysfunctional. T cells that enter the tumor often become exhausted, with reduced proliferative capacity, diminished cytokine secretion, and sustained expression of inhibitory receptors [35,36,37,38]. In parallel, poor antigen presentation and defective dendritic-cell maturation prevent the efficient initiation of new antitumor responses [58,59,60,61]. This means the tumor can both block new immune recruitment and neutralize the immune cells already present [62,63].

Stromal and vascular barriers. The physical organization of the tumor also contributes strongly to immune suppression. Abnormal vasculature limits lymphocyte trafficking, and stromal remodeling creates a dense, often collagen-rich matrix that traps immune cells in peritumoral regions rather than allowing them to penetrate the malignant core [40,64,65]. Cancer-associated fibroblasts can reinforce this exclusion through secretion of chemokines, matrix components, and suppressive factors [36,38,61]. These structural features matter because even a vaccine-primed T-cell response can fail if the effector cells cannot reach the tumor parenchyma.

Relevance for therapy. This immunosuppressive environment explains why many immunotherapies work only in selected patients or in combination regimens. Checkpoint blockade, vaccines, oncolytic viruses, and adoptive cell therapies all face the same barrier: the tumor microenvironment can neutralize immune activation at multiple levels simultaneously [37,66,67,68]. For therapeutic cancer vaccines specifically, the major challenge is not simply generating tumor-specific T cells, but ensuring those T cells can survive, traffic, and function within a metabolically deprived and suppressive tumor niche [69]. That is why modern strategies increasingly aim to combine immune activation with microenvironmental reprogramming [27,70,71,72,73,74,75,76].

In short, the immunosuppressive environment in cancer is a multilayered system of cellular, molecular, metabolic, and structural barriers that collectively prevent productive antitumor immunity. It is not a side effect of cancer biology; it is one of the central mechanisms by which tumors persist and progress. On this basis, this review systematically discusses cold-to-hot conversion strategies, combination regimens, biomarker-guided patient stratification, and current translational challenges, with the goal of providing a framework for advancing precision cancer vaccine therapy.

## 2. Inhibition of Cancer Vaccine Efficacy by the Tumor Microenvironment

The immunosuppressive TME dampens the priming and amplification of vaccine-induced immunity through multiple layers: physical barriers [64], immunosuppressive cell networks [77], an imbalance of soluble factors [78,79], metabolic dysregulation [80], and defective dendritic cell antigen presentation [81]. Moreover, the immune-desert and immune-excluded TMEs differ significantly in their inhibitory patterns and key targets [35,37,82].

### 2.1. Physical Barriers: Impeding Immune Cell Infiltration and Vaccine Delivery

Many of the mechanistic insights into tumor physical barriers have been derived from studies of pancreatic ductal adenocarcinoma (PDAC), a malignancy characterized by an exceptionally dense desmoplastic stroma rich in extracellular matrix components. PDAC is often considered an extreme but highly instructive model for studying physical immune exclusion. However, it must be emphasized that PDAC represents a unique tumor entity, and while similar physical barriers exist in other solid tumors—such as breast, lung, and colorectal carcinomas—their extent and composition vary considerably. Therefore, findings from PDAC should be applied to other cancer types with caution.

Excessive cross-linking and deposition of extracellular matrix (ECM), along with malformed tumor vasculature and disturbed blood perfusion, collectively create rigid physical barriers within the tumor lesion [64]. Cancer-associated fibroblasts (CAFs) secrete large amounts of collagen, proteoglycans, and hyaluronic acid, which increase tissue stiffness and interstitial pressure, thereby hindering the penetration of nanoparticles, lipid carriers, and viral vectors [32,39,40,41]. Abnormal tumor vessels exhibit features such as defective walls, aberrant permeability, and heterogeneous, sparse distribution, further reducing the delivery efficiency of vaccine antigens and circulating immune cells to the tumor site [30,39,42].

In the immune-excluded TME, the number of immune cells is not significantly reduced, but they are confined to the peritumoral stromal area by dense matrix and cannot enter the tumor core to exert their killing function. CAFs specifically interfere with T-cell chemotactic signals by overexpressing CXCL12, thereby further consolidating the immune-excluded phenotype [36,38,43].

### 2.2. Inhibiting Vaccine-Induced Immune Activation

Within the TME, regulatory T cells (Tregs), M2-polarized tumor-associated macrophages (TAMs), myeloid-derived suppressor cells (MDSCs), and CAFs form an interactive immunosuppressive network [43,44,45,46,47,48]. Tregs suppress effector T-cell proliferation, activation, and cytotoxicity through direct cell–cell contact or secretion of inhibitory factors such as TGF-β and IL-10 [49,50,51]. M2-type TAMs, which dominate the macrophage population in the TME, not only secrete abundant immunosuppressive factors that block dendritic cell maturation and antigen presentation but also release matrix-remodeling enzymes that promote ECM deposition and angiogenesis [83]. MDSCs, through the production of reactive oxygen species, nitric oxide, and arginase, deplete essential metabolites required for local immune responses, thereby broadly inhibiting the functions of T cells and dendritic cells; they are key mediators of vaccine resistance in the cold tumor microenvironment [16,43]. Moreover, cancer stem cells can modulate the phenotype of stromal and immune cells via paracrine signals, further amplifying local immunosuppression [48]. In glioblastoma, TAMs are predominant and exhibit an M2 phenotype, with tumor-associated macrophages and MDSCs being the major immunosuppressive populations [41,84]. Similar enrichment of immunosuppressive cells has been observed in various other solid tumors, including head and neck cancer, ovarian cancer, sarcoma, hepatocellular carcinoma, and pediatric rhabdomyosarcoma [85,86,87,88,89,90].

### 2.3. Soluble Suppressive Factors and Metabolic Dysregulation: Impairing Immune Cell Function

Tumor cells and stromal cells continuously secrete inhibitory cytokines such as TGF-β, IL-10, and IL-6, which block the maturation and differentiation of dendritic cells and downregulate the expression of co-stimulatory and MHC molecules [43,52]. The Warburg effect-driven metabolic reprogramming of tumor cells, combined with a hypoxic tumor microenvironment, results in multiple metabolic-immune suppressive mechanisms: hypoxia activates the HIF-1α pathway, upregulating the transcription of immunosuppressive and angiogenic factors; massive lactate accumulation lowers local pH, directly inhibiting effector T-cell proliferation and cytotoxic activity [43,53,54,55,56]. The adenosine pathway becomes a metabolic immune checkpoint in hypoxic tumors, where limited oxygen and high extracellular ATP turnover drive accumulation of extracellular adenosine. This adenosine is generated largely through hypoxia-responsive nucleotide catabolism, especially via ectonucleotidase activity, and it rises to levels that are sufficient to reshape local immune behavior rather than merely reflect tissue stress. Once present in the tumor microenvironment, adenosine transmits inhibitory signals mainly through A2A and A2B receptors on immune cells. These receptors couple to cAMP-elevating pathways that suppress T-cell receptor signaling, reduce cytokine production, impair cytotoxic function, and blunt antigen-presenting cell activity. As a result, the earliest phases of antitumor immunity are weakened: dendritic-cell priming becomes less effective, effector T cells are harder to activate, and NK-cell responses are dampened before a productive immune attack can be established [57].

### 2.4. Defective Antigen Presentation: Compromising Vaccine-Induced Immune Activation

Dendritic cells are the central professional antigen-presenting cells for initiating systemic anti-tumor immunity in response to cancer vaccines, but the immunosuppressive TME can induce DC maturation defects, functional impairment, and increased apoptosis. High levels of TGF-β, VEGF, and other inflammatory inhibitory factors expressed in the TME significantly downregulate the expression of MHC-I/II, CD80, and CD86 on the DC surface, thereby impairing antigen uptake, processing, and cross-presentation [84,85,86,87]. Furthermore, tumor cells reduce overall immunogenicity by downregulating their own antigen expression, losing HLA molecules, and secreting immune-escape-related molecules, which further weakens the recognition and clearance efficiency of vaccine-induced immune cells [62,63].

### 2.5. Distinct Inhibitory Mechanisms of Immune-Desert and Immune-Excluded Tumor Microenvironments

**Figure 1:**
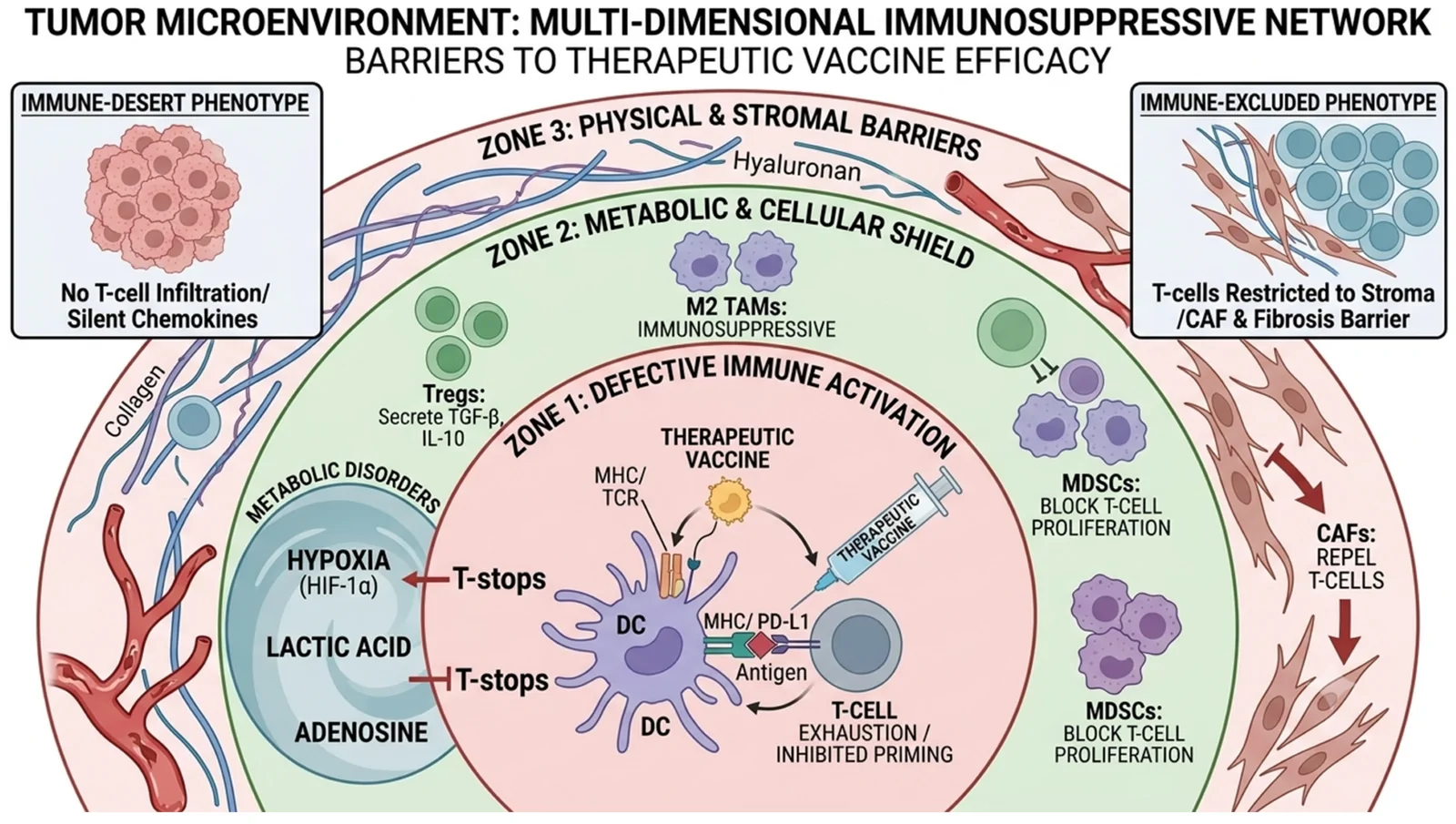
Tumor microenvironment as a multidimensional immunosuppressive network that limits therapeutic vaccine efficacy. The tumor microenvironment is composed of malignant cells, stromal elements, suppressive immune populations, abnormal vasculature, and soluble mediators that together create a barrier to effective anti-tumor immunity. These components restrict antigen presentation, impair dendritic cell function, inhibit effector T-cell priming and infiltration, and promote T-cell exhaustion, thereby blunting vaccine-induced immune responses. In parallel, immunosuppressive cytokines, metabolic competition, hypoxia, and extracellular matrix remodeling further reinforce local immune evasion. Collectively, these mechanisms form a coordinated suppressive niche that surrounds the tumor and prevents therapeutic vaccines from generating durable anti-tumor immunity. This conceptual schematic was generated using Google Gemini 3.

The immune-desert TME is characterized by a pronounced lack of effector T cells and mature dendritic cells within the tumor parenchyma, accompanied by silenced local chemokine expression, blocked lymphatic homing pathways, and deficient co-stimulatory signals, making it difficult for vaccines to prime effective immune responses [35,36,37,38]. In contrast, the immune-excluded TME does not show an obvious paucity of immune cells, but dense extracellular matrix and abnormal blood vessels form physical barriers that prevent vaccine-activated effector T cells from penetrating into the tumor parenchyma, leaving them trapped in the peritumoral area [40,65]. The differences in cellular composition, molecular pathways, and inhibitory characteristics between these two phenotypes provide an important basis for the precision design and individualized adaptation of vaccine combination regimens [37,38] (Figure 1).

## 3. Remodeling of the Tumor Microenvironment Driven by Cancer Vaccines

Rather than passively succumbing to TME immunosuppression, different types of cancer vaccines can actively reverse the suppressive microenvironment and promote the transition from cold to immunologically inflamed tumors by activating innate immune signals, regulating the secretion of inflammatory factors, reshaping the polarization balance of myeloid cells, restoring chemokine gradients, and improving vascular and stromal structures [91,92].

### 3.1. Activating and Recruiting Effector Immune Cells into Tumors

Cancer vaccines efficiently deliver tumor antigens to dendritic cells in the lymph nodes and within the TME, promoting DC maturation and activation, initiating CD4^+^ helper T-cell and CD8^+^ cytotoxic T-cell responses, and establishing long-term immunological memory [93,94,95,96]. In the study by Wang et al., an ultrasound-driven nanovaccine (G5-CHC-R) increased CD8^+^ T-cell infiltration to 15.5% in a pancreatic cancer model, accompanied by enhanced IFN-γ secretion [91]. Activated effector T cells secrete chemokines such as CXCL9, CXCL10, and CXCL11, thereby re-establishing chemokine gradients within the TME that guide the directional infiltration of peripheral effector T cells into the tumor lesion [97,98]. Photodynamic therapy combined with nanoparticles can also induce immunogenic cell death (ICD), converting the tumor into an in situ vaccine [99].

### 3.2. Promoting DC Maturation and Antigen Cross-Presentation

Adjuvant-formulated cancer vaccines are cancer vaccines that include an immune-stimulating adjuvant to make the body mount a stronger, broader, and more durable anti-tumor response. In cancer patients, vaccines often need adjuvants because tumors create an immunosuppressive environment that weakens antigen presentation and T-cell activation. An adjuvant helps the vaccine do more than just deliver antigen. It activates innate immune sensors, promotes dendritic cell maturation, improves antigen processing and presentation, and increases the activation and expansion of tumor-specific T cells. Adjuvant-formulated cancer vaccines comprehensively promote dendritic cell maturation and upregulate the expression of MHC molecules and co-stimulatory molecules by activating pattern recognition receptor signaling pathways such as TLR, STING, and NF-κB [100,101]. Mature DCs efficiently cross-present exogenous vaccine antigens and activate polyclonal CD8^+^ T-cell responses. In multiple models, the nanovaccine G5-CHC-R combined with whole-body ultrasound promoted DC maturation: in an orthotopic pancreatic cancer model, the proportion of mature DCs (CD11c^+^CD80^+^CD86^+^) within tumors reached 40.3%, and that in lymph nodes reached 17.9%; in a spontaneous breast cancer (4T1) lung metastasis model, the proportion of mature DCs within tumors increased approximately 2.6-fold, lymph node mature DCs also increased, and CD8^+^ T-cell infiltration increased approximately 2.9-fold [91]. A STING agonist is a molecule that activates the STING (Stimulator of Interferon Genes) pathway, a key component of the innate immune system that detects cytosolic DNA and triggers strong antiviral and anti-tumor immune responses. STING (also known as TMEM173 or MITA) is a protein in the endoplasmic reticulum of many cell types, including immune cells, stromal cells, and some tumor cells. It acts as a sensor for foreign or misplaced DNA in the cell’s cytoplasm (e.g., from viruses, bacteria, or damaged/dying tumor cells). When cytosolic DNA is detected, the enzyme cGAS produces the natural second messenger 2′3′-cGAMP (a cyclic dinucleotide, or CDN). cGAMP binds to and activates STING, which then triggers a signaling cascade involving TBK1 and IRF3. This leads to the production of type I interferons (e.g., IFN-β), other pro-inflammatory cytokines, and chemokines that recruit and activate T cells, natural killer cells, and other immune effectors. The pathway bridges innate and adaptive immunity and helps “alert” the immune system to threats like infections or cancer. STING agonists are synthetic or modified compounds designed to mimic natural ligands like cGAMP and directly or indirectly activate STING. They amplify immune responses, particularly useful in cancer immunotherapy: they can turn “cold” tumors (with low immune infiltration) into “hot” ones by promoting inflammation, antigen presentation, and T-cell priming within the tumor microenvironment. They are often studied in combination with checkpoint inhibitors (e.g., anti-PD-1/PD-L1). Types of STING agonists include: (1) Cyclic Dinucleotide (CDN) analogs—direct mimics of cGAMP or bacterial CDNs (e.g., c-di-GMP), such as ADU-S100 (MIW815), MK-1454, and BMS-986301, which are potent but face challenges like poor stability, rapid degradation, or limited systemic delivery; (2) Non-CDN small molecules—more drug-like compounds with better stability and pharmacokinetics, such as diABZI and MSA-2; and (3) Advanced delivery formats—antibody-drug conjugates (ADCs) linking STING agonists to tumor-targeting antibodies, as well as exosomes, nanoparticles, or engineered bacteria (e.g., SYNB1891) for targeted or sustained release. Many STING agonists have shown promising preclinical anti-tumor activity and are in clinical trials (mostly Phase 1/2), often given intratumorally initially to limit systemic toxicity (e.g., cytokine release). Challenges include optimizing delivery, reducing side effects, and identifying responsive patients. Co-administration of a STING agonist can further amplify DC cross-presentation and repair the antigen-presentation defects of the immune-desert TME [102].

### 3.3. Reshaping the Myeloid Cell Landscape in the Tumor Microenvironment

Cancer vaccines can reshape the polarization and differentiation patterns of myeloid cells in the TME by balancing local pro-inflammatory and anti-inflammatory factor secretion. Pro-inflammatory cytokines such as IFN-γ and TNF-α induced by vaccination drive M2-type macrophages to polarize toward the anti-tumor M1 phenotype, while simultaneously inhibiting the local recruitment and expansion of MDSCs [103]. In a study by Zhang et al., a Listeria-vectored cervical cancer vaccine reduced the expression of MDSC-inducing factors through inhibition of the JAK1-STAT1 and JAK2-STAT3 pathways, thereby decreasing MDSC formation and impairing their immunosuppressive function [104]. After inoculation of advanced tumors with the PC7A nanovaccine, infiltration of polymorphonuclear myeloid-derived suppressor cells (PMN-MDSCs) increased, and their immunosuppressive activity was enhanced; CD300ld is a cell-surface immune receptor in the CD300 family, also known as CD300D or CMRF35A4. It is a single-pass type I membrane protein with an Ig-like domain, and in mice, it is strongly associated with neutrophils, especially polymorphonuclear myeloid-derived suppressor cells (PMN-MDSCs) in tumors. Functionally, recent studies link CD300ld to immune regulation in two different contexts: in cancer, it supports tumor-promoting neutrophil/PMN-MDSC activity and helps create an immunosuppressive microenvironment, while in infection it appears to promote neutrophil bacterial phagocytosis. In the tumor setting, blocking CD300ld reduced tumor growth and improved response to anti-PD-1 therapy in mouse models. 

In short, CD300ld is best thought of as a neutrophil-associated immunoregulatory receptor with emerging relevance in both cancer immunology and innate defense. Accordingly, knockout of CD300ld abrogated PMN-MDSC recruitment and T-cell suppressive function, restoring vaccine efficacy [105].

### 3.4. Improving Vascular Normalization and Reducing Extracellular Matrix Deposition

Personalized neoantigen vaccines and peptide vaccines can inhibit aberrant angiogenesis and promote the normalization of tumor vascular structure and function by modulating the TME cytokine network [106,107]. In a pancreatic cancer model treated with the anti-gastrin vaccine PAS combined with anti-PD-1, Masson’s trichrome staining showed a significant reduction in the fibrosis score in the combination group (*p* = 0.0009), indicating that the vaccine reduced ECM deposition [108]. Cancer vaccines can also inhibit excessive proliferation and activation of CAFs, reduce tumor tissue stiffness and interstitial pressure, break physical barriers, and thereby create favorable conditions for extensive infiltration of effector immune cells [109,110].

## 4. Strategies for Cold-to-Hot Tumor Conversion Based on Bidirectional Crosstalk

Leveraging the bidirectional regulatory relationship between cancer vaccines and the TME, multidimensional interventions–targeting immunosuppressive cells, correcting metabolic disorders, breaking physical barriers, and optimizing vaccine design with adjuvants and carriers–can precisely reverse the immunosuppressive landscape and efficiently achieve the phenotypic conversion from cold to hot tumors [36,37,65,111].

### 4.1. Targeting Immunosuppressive Cells in the Tumor Microenvironment

Specific targeted drugs that deplete or functionally inhibit Tregs, M2-type macrophages, and MDSCs can effectively relieve the immunosuppressive constraints of the TME [112,113,114]. CTLA-4 antibody (ipilimumab) depletes local Tregs in an Fc-dependent manner. However, high expression of the inhibitory Fc receptor FcγRIIB on tumor-infiltrating myeloid cells (e.g., tumor-associated macrophages and dendritic cells) in the TME limits antibody-dependent cellular cytotoxicity/phagocytosis (ADCC/ADCP), thereby reducing Treg depletion efficacy; Fc engineering can enhance Treg depletion [115]. Anti-CD25 antibody (Fc-optimized, IL-2-retaining) combined with PD-1 blockade increases the CD8^+^/Treg ratio [116]. Targeting the IL-8/CXCR2 axis with AZD5069 eliminates MDSC activity and sensitizes tumors to anti-PD-L1 [117]. In pancreatic cancer, CXCR2-targeted nanoparticle delivery of JAK2/STAT3 inhibitors suppresses arginase activity and reduces tumor burden, representing a novel strategy to specifically disrupt MDSC-mediated immunosuppression [118]. A Listeria-vectored cervical cancer vaccine reduces MDSC formation via inhibition of the JAK-STAT signaling pathway, significantly lowering MDSC levels and function in vivo, which is a key step in dismantling TME immunosuppression [104]. CSF-1R inhibitors (e.g., PLX3397) block M2-type macrophage polarization [119,120]. In the LLC1 lung cancer “cold” tumor model, a neoantigen DC vaccine combined with CpG and anti-CD38 antibody significantly reduced intratumoral Tregs and inhibited tumor growth [26]. A sequential combination of immunosuppressive cell targeting with tumor vaccines can produce strong synergistic effects [121,122].

### 4.2. Modulating Tumor Microenvironment Metabolism and Breaking Physical Barriers

Targeting features such as TME hypoxia, lactate accumulation, and aberrant adenosine enrichment, agents such as adenosine receptor antagonists (e.g., AZD4635) and lactate metabolism modulators—including LDH or MCT inhibitors, which can starve tumor cells of metabolic flexibility, reduce acid-driven invasion, and weaken lactate-mediated immune suppression in the tumor microenvironment—can reverse metabolic-mediated immunosuppression. Some strategies combine lactate blockade with other metabolic stressors, such as intracellular acidification or oxygen modulation, to amplify antitumor effects [57,123,124]. Using PEGPH20 to degrade hyaluronic acid or FAK inhibitors to reduce matrix stiffness breaks physical barrier constraints from the stromal architecture [125,126,127,128]. For the immune-excluded TME, it is necessary to first relieve physical barriers through matrix degradation and anti-angiogenic interventions, followed by sequential administration of cancer vaccines. For the immune-desert TME, antigen presentation and chemokine signals must first be restored (e.g., using FLT3L or CD40 agonists) before initiating vaccination [129,130,131,132,133].

### 4.3. Optimizing Vaccine Design to Enhance Tumor Microenvironment Remodeling Capacity

**Figure 2:**
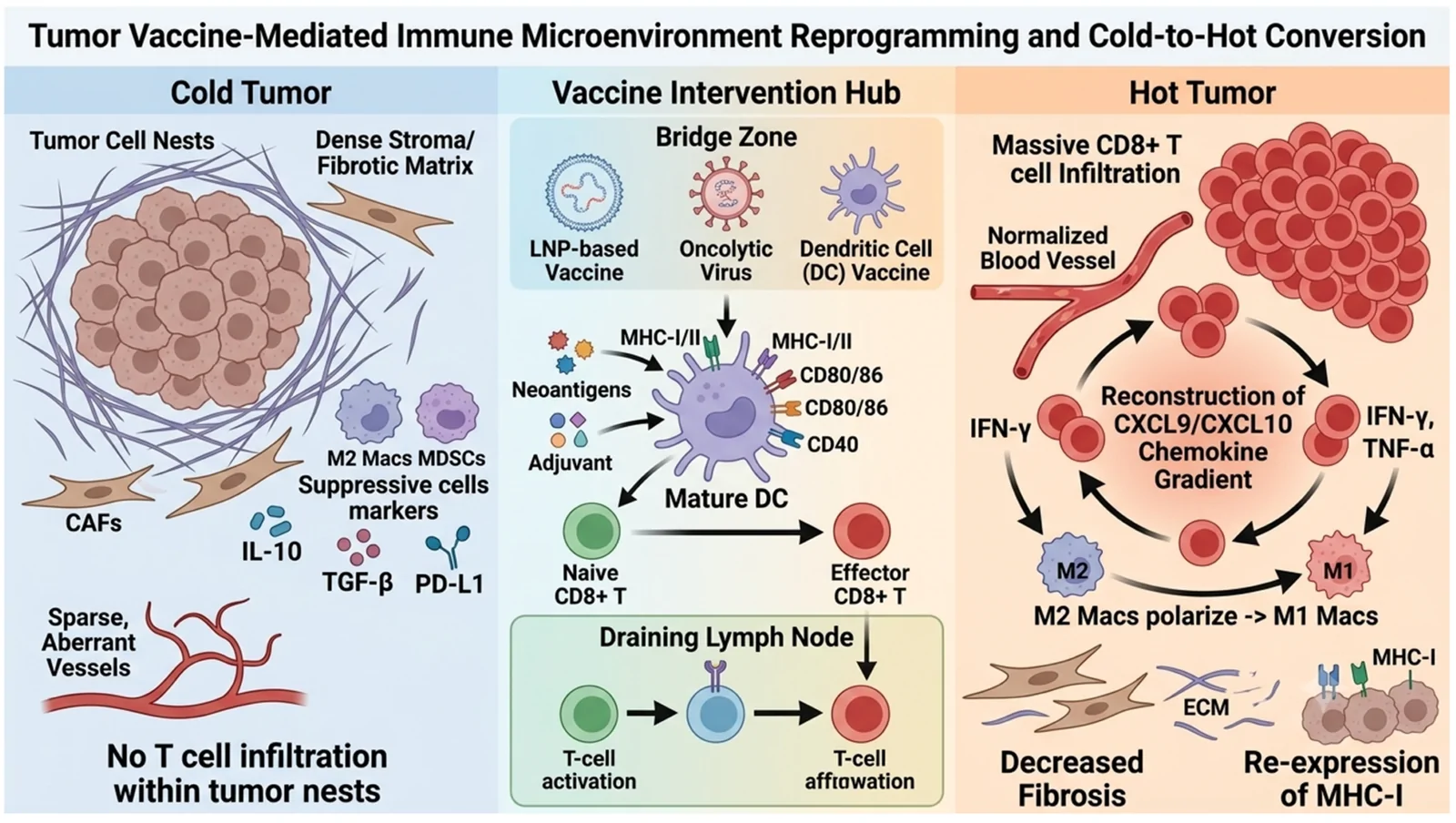
Tumor vaccine-mediated immune microenvironment reprogramming and cold-to-hot conversion. This conceptual schematic was generated using Google Gemini 3.

Screening high-affinity neoantigens based on tumor mutational burden, HLA typing, and TME immunophenotype improves vaccine specificity and immune activation strength [134,135]. Optimizing lipid nanoparticles, cell-membrane biomimetic carriers, and exosome delivery systems enables lymph node-targeted and TME-targeted enrichment of antigens [136,137,138]. Combining multifunctional adjuvants such as TLR (CpG, R848) and STING (ADU-S100) agonists simultaneously activates innate and adaptive immunity, releasing the local immunosuppressive brakes of the TME [139,140,141,142]. In glioblastoma patients, the neoantigen vaccine (GAPVAC) induced robust neoantigen-specific T-cell responses in those not receiving dexamethasone, whereas those on dexamethasone showed no response, indicating that immune status is critical for vaccine-driven TME remodeling [143] (Figure 2).

## 5. Combination Strategies to Enhance Vaccine Efficacy

Single-agent cancer vaccines are limited by TME heterogeneity, multiple immunosuppressive mechanisms, physical barriers in solid tumors, and insufficient antigen presentation, making it difficult to achieve ideal clinical efficacy. Sequential or simultaneous combination of cancer vaccines with immune checkpoint inhibitors, TME modulators, chemoradiotherapy, oncolytic viruses, and novel delivery systems enables multi-target, multi-timing synergistic regulation of the TME [144,145,146,147,148].

### 5.1. Cancer Vaccines Combined with Immune Checkpoint Inhibitors

Cancer vaccines prime and expand antigen-specific effector T cells, while PD-1/PD-L1 and CTLA-4 inhibitors relieve T-cell exhaustion and immunosuppressive brakes [146,149,150,151]. In a phase Ib trial of the GVAX pancreatic cancer vaccine combined with ipilimumab, more patients in the combination arm showed expansion of mesothelin-specific CD8^+^ T cells, with a trend toward prolonged median overall survival [16]. In melanoma, the oncolytic virus vaccine T-VEC plus ipilimumab achieved an objective response rate (ORR) of 39% (vs. 18% for ipilimumab alone) [152]. In non-small cell lung cancer, durvalumab maintenance therapy after chemoradiotherapy (PACIFIC trial) significantly improved progression-free survival (16.8 vs. 5.6 months) [153]. In hepatocellular carcinoma, atezolizumab plus bevacizumab (IMbrave150) has become a first-line standard [154,155]; the dual immune checkpoint inhibitor combination durvalumab plus tremelimumab was also approved based on the HIMALAYA trial [156,157]. Vaccine-mediated TME remodeling can upregulate PD-L1 expression in the tumor lesion, thereby increasing sensitivity to immune checkpoint inhibitors, while immune checkpoint blockade further promotes deep infiltration of effector T cells into the tumor [158]. Clinical studies have confirmed that this combination approach improves ORR and progression-free survival in various solid tumors [159,160]; however, immune-related adverse events (irAEs) show dose- and timing-dependency, requiring individualized regimen optimization [161,162].

### 5.2. Cancer Vaccines Combined with TME Modulators

Anti-angiogenic agents (e.g., bevacizumab, cabozantinib) promote tumor vascular normalization, improving tissue perfusion and deep delivery of vaccines and immune cells [163,164]. In the COSMIC-021 trial, cabozantinib plus atezolizumab achieved an ORR of 32% in metastatic castration-resistant prostate cancer [165]. TME modulators such as metabolic regulators, immunosuppressive cell-targeting drugs, and inflammatory pathway modulators can relieve local immunosuppression through metabolic reprogramming, cell phenotype regulation, and inflammatory balance modulation [166,167]. In a pancreatic cancer model, the anti-gastrin vaccine PAS, combined with anti-PD-1, significantly reduced fibrosis and M2-type tumor-associated macrophages [108,168]. The CD40 agonist sotigalimab, combined with chemotherapy and nivolumab, achieved a 1-year overall survival rate of nearly 60% in pancreatic cancer [169]. HDAC3 inhibitors can enhance immunotherapy through the cGAS-STING and ferroptosis pathways [170]. Vagus nerve stimulation, as a non-pharmacological physical intervention, has the potential to convert cold glioblastoma into hot glioblastoma by inhibiting IL-6 [130].

### 5.3. Cancer Vaccines Combined with Radiotherapy/Chemotherapy

Radiotherapy and chemotherapy directly kill tumor cells, induce immunogenic cell death (ICD), release large amounts of tumor antigens and damage-associated molecular patterns (DAMPs), and thereby create an in situ vaccine effect [171,172]. Radiotherapy combined with a CTLA-4 inhibitor induced an abscopal effect in lung cancer, with shrinkage of non-irradiated lesions by ≥30% [173]. In KRAS-mutant pancreatic cancer, the ELI-002 vaccine (amphiphile-modified G12D/G12R long peptides plus CpG) combined with chemotherapy induced T-cell responses and biomarker clearance in a phase I trial [174,175]. Different radiotherapy fractionation regimens, chemotherapy drug types, and administration timing significantly affect the response and safety of combination therapy [97,176,177]. Type I T cells sensitize refractory tumors to chemotherapy by secreting IFN-γ to induce SOCS1, which inhibits oncogenic signaling pathways [97].

### 5.4. Cancer Vaccines Combined with Oncolytic Viruses

Oncolytic viruses specifically lyse tumor cells, release tumor antigens, and secrete pro-inflammatory factors, actively remodeling the local immunosuppressive microenvironment [22,178,179]. T-VEC (HSV-1 expressing GM-CSF) is the first FDA-approved oncolytic virus vaccine; in melanoma, it significantly increased ORR when combined with ipilimumab [152,178]. In pancreatic cancer, LOAd703 (an oncolytic adenovirus expressing 4-1BBL and CD40L) combined with chemotherapy and atezolizumab achieved an ORR of 44% and a disease control rate of 94% in 10 patients [180]. The combination of oncolytic viruses and cancer vaccines can doubly enhance antigen presentation and immune cell activation, restore chemokine gradients, and break the intrinsic immune escape pattern of tumors, making it particularly suitable for “cold” tumors [181,182]. In addition to engineered oncolytic viruses, an emerging strategy involves the repurposing of licensed viral vaccines—originally developed for infectious disease prevention—as off-the-shelf anti-tumor immunotherapies. Preclinical studies have demonstrated that vaccines such as measles, mumps, rubella (MMR), yellow fever, Japanese encephalitis, Zika, rabies, influenza, rotavirus, modified vaccinia Ankara (MVA), and dengue vaccines all exhibit oncolytic or immunomodulatory activity against various human cancer cell lines. Similar to oncolytic viruses, these licensed vaccines can reshape the immunosuppressive tumor microenvironment—driving “cold” tumors toward an immune-active (“hot”) state—and synergize with immune checkpoint inhibitors. Mechanistically, live-attenuated vaccines can exert direct oncolytic effects through viral replication, while both live and inactivated vaccines contribute to TME remodeling via type I interferon signaling, upregulation of PD-L1, and recruitment of dendritic cells and CD8^+^ T cells. Importantly, pre-existing immunity to these widely used vaccines does not necessarily impair their antitumor efficacy and may, in some cases (e.g., yellow fever vaccine), even enhance tumor clearance. Clinically, intratumoral injection of the attenuated measles vaccine strain (MV-EZ) has been shown to induce tumor infection and oncolysis in patients with cutaneous T-cell lymphoma and recurrent breast cancer. Given their well-established safety profiles, global availability, and cost-effectiveness, repurposed licensed viral vaccines represent a readily accessible and promising addition to the cancer immunotherapy arsenal, although further randomized controlled trials are warranted to optimize delivery routes and combination regimens [183].

### 5.5. Cancer Vaccines Combined with Novel Delivery Systems

Novel delivery systems such as lipid nanoparticles [184], cell-membrane biomimetic carriers [185], exosomes [186], and peptide-based composite carriers [187] improve the in vivo stability of tumor antigens, their ability to target lymph nodes, and the efficiency of dendritic cell uptake [188,189]. In the study by Wang et al., the G5-CHC-R nanovaccine (constructed based on G5 PAMAM, particle size ~12 nm) exhibited excellent deep penetration ability in a 3D pancreatic cancer tumor spheroid model, delivering sonosensitizers and immune modulators to the tumor core [91]. Integrated design of the delivery system with vaccine antigens and adjuvants enables targeted presentation and controlled release [92,99,111,190]. For example, FeAu-MB complexes self-assemble in the tumor microenvironment and induce ICD when combined with photothermal therapy, converting “cold” tumors into “hot” tumors [191]. Bioorthogonally activated sonodynamic therapy can induce tumor-specific pyroptosis and enhance the in situ vaccine effect [92].

## 6. Biomarkers for Predicting Vaccine Response and Patient Stratification

The efficacy of cancer vaccines shows substantial individual variability, which is closely related to TME immunophenotype, tumor mutational burden, HLA typing, and baseline host immune status [16,88,192,193,194,195].

### 6.1. Tumor Microenvironment-Related Biomarkers

The infiltration density and spatial distribution of CD8^+^ effector T cells and mature dendritic cells within tumor tissues are positively correlated with the response to cancer vaccine therapy; conversely, high infiltration of Tregs, M2-type macrophages, and MDSCs, together with a disordered chemokine profile, indicate a significantly elevated risk of vaccine resistance [34,132,196]. In a glioblastoma neoantigen vaccine trial, patients who did not receive dexamethasone showed an average increase of 71 CD8^+^ T cells/mm^2^ in recurrent tumors [143]. The TME classification system based on immune-desert, immune-excluded, and inflamed (hot) phenotypes can directly guide patient selection for therapeutic regimens and predict long-term prognosis [37,66,197,198].

### 6.2. Tumor Immunogenicity-Related Biomarkers

Tumor mutational burden (TMB), neoantigen quantity, and HLA typing compatibility are core indicators of intrinsic tumor immunogenicity. dMMR/MSI-H colorectal cancer is highly sensitive to pembrolizumab, with an ORR of 40% [192,199]. In pancreatic cancer, the KRAS G12D mutation can serve as a neoantigen target, and TCR-T cells achieved a 72% partial response in one patient [16,200]. Baseline expression levels of tumor-associated antigens and tumor-specific antigens, combined with mutational spectrum characteristics, enable the precise selection of patient populations that are most likely to benefit from cancer vaccines [135,201].

### 6.3. Host Immune Status-Related Biomarkers

The distribution of peripheral blood immune cell subsets, the functional homeostasis of dendritic cells, baseline expression of immune checkpoint molecules, and cytokine profiles all directly influence the immune-activation efficacy of cancer vaccines [202,203,204]. Gut microbiota composition regulates systemic immune homeostasis; Akkermansia muciniphila and Bifidobacterium adolescentis are positively associated with response to immune checkpoint inhibitors [193,205,206]. The use of glucocorticoids such as dexamethasone severely impairs vaccine-induced T-cell responses [207]. Fecal microbiota transplantation (FMT) can improve responses in PD-1-resistant melanoma [204,208,209]. Molecules related to trained immunity (e.g., BCG- or β-glucan-induced epigenetic reprogramming) may also serve as emerging biomarkers for predicting vaccine responsiveness [195,210,211].

### 6.4. Clinical Application of Biomarkers

**Figure 3:**
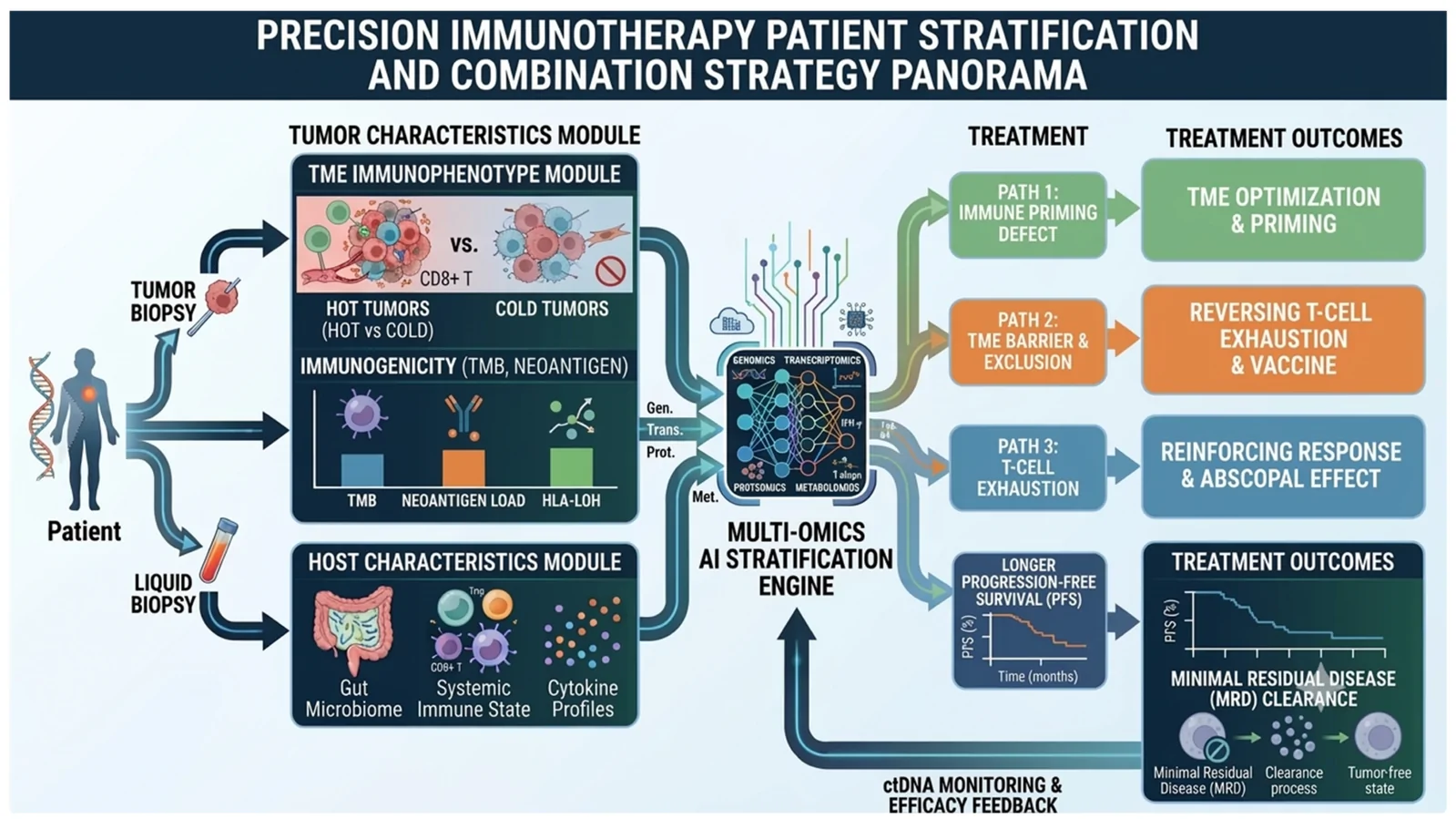
Precision immunotherapy patient stratification and combination strategy panorama. This conceptual schematic was generated using Google Gemini 3.

The predictive power of a single biomarker is limited; combining multiple indicators such as TME immunophenotype, TMB, HLA typing, and host immune status significantly improves the accuracy of patient stratification [16,212]. With the widespread adoption of single-cell RNA sequencing, spatial transcriptomics, and multi-omics integration technologies, more novel molecular and cellular biomarkers will be discovered, further supporting the individualized clinical application of cancer vaccines [193,213,214] (Figure 3).

## 7. Clinical Trials and Translational Challenges

### 7.1. Overview of Clinical Trials

In recent years, the number of clinical trials of cancer vaccines as single agents or in multimodal combination therapies has continued to increase. Neoantigen mRNA vaccines (autogene cevumeran, mRNA-4157) combined with PD-1 inhibitors have been shown to prolong progression-free survival in solid tumors such as pancreatic cancer and melanoma [15,215]. The CLDN18.2-targeted CAR-T cell therapy (CT041) enrolled 37 patients with advanced CLDN18.2-positive gastric or pancreatic cancer in a phase I trial. In the pancreatic cancer subgroup (n = 12), one partial response (PR) and one complete response (CR) were observed, with an objective response rate (ORR) of 16.7% [16,216]. In hepatocellular carcinoma, atezolizumab plus bevacizumab and durvalumab plus tremelimumab have been approved as first-line treatments [154,155]. In gastric cancer, nivolumab plus chemotherapy (CheckMate 649) has become a first-line standard [217]. In microsatellite-stable (MSS) colorectal cancer, the combination of immune checkpoint inhibitors with chemotherapy plus anti-VEGF (AtezoTRIBE) improved overall survival [218]. Currently, most vaccine combination regimens are still in phase I/II exploratory stages, lacking large-scale, multicenter, phase III evidence-based data, and issues such as administration timing, dose ratio, and applicable indications require further standardization [16,174,219,220].

### 7.2. Translational Challenges

The clinical translation of cancer vaccines faces multiple practical bottlenecks: (i) pronounced spatiotemporal heterogeneity of the TME, leading to large differences in efficacy of the same vaccine regimen across different pathological types and immune phenotypes of tumors [221,222,223]; (ii) the lack of a standardized, generalizable multi-omics biomarker system [224,225]; (iii) insufficient deep penetration and targeted delivery capacity of vaccines in solid tumors [226,227]; (iv) the propensity of multimodal combination therapy to induce immune-related adverse events (e.g., CRS, colitis, myocarditis), with grade ≥3 irAEs for dual immune checkpoint inhibitor combinations reaching 42–53% [228,229,230]; (v) the complexity of mutation screening, carrier preparation, and quality control for individualized neoantigen vaccines, with a production lead time of about 3–4 months, which limits widespread clinical application [225,231,232]; and (vi) limitations of animal models, as existing models cannot fully recapitulate the human TME [233,234].

### 7.3. Strategies to Overcome Translational Challenges

In the future, it will be necessary to build standardized biomarker evaluation systems using multi-omics technologies [235,236]; to continuously optimize vaccine antigen screening algorithms (e.g., HLAthena), multifunctional adjuvants, and intelligent nano-delivery carriers [188]; to conduct large-scale, multicenter clinical trials that optimize the timing of combination therapy (e.g., vaccine first, then immune checkpoint inhibitor) [237]; to simplify individualized vaccine preparation and quality control processes (e.g., shortening the production time of mRNA platforms to around 6–8 weeks) [238,239]; to establish comprehensive monitoring and management systems for immune-related adverse events [240]; and to use artificial intelligence to assist neoantigen prediction and digital twin models for vaccine design [5,241,242], thereby pushing cancer vaccines from basic research toward routine clinical application from all directions.

## 8. Conclusions and Future Perspectives

### 8.1. Conclusions

There is a close and highly dynamic bidirectional crosstalk between cancer vaccines and the tumor microenvironment (TME), in which each continuously influences the effectiveness of the other. Therapeutic cancer vaccines are intended to induce tumor-specific immunity by enhancing antigen presentation, priming cytotoxic T lymphocytes, and promoting durable immune memory. However, the success of this process depends not only on the quality of the vaccine platform and antigen selection, but also on the local immune context in which the response develops. The TME often functions as a complex suppressive ecosystem that limits vaccine efficacy through multiple, overlapping mechanisms, including defective dendritic cell maturation, poor antigen processing, impaired T-cell trafficking, exclusion of effector lymphocytes, and induction of T-cell exhaustion [16].

At the same time, a successful vaccine response can actively reshape the TME. By increasing the frequency and activity of tumor-reactive T cells, enhancing interferon signaling, and stimulating innate and adaptive immune pathways, vaccines can convert a suppressive niche into a more inflamed and immunologically permissive environment [39,91]. This remodeling may improve immune cell infiltration, increase antigen visibility, and weaken the dominance of suppressive populations such as regulatory T cells, tumor-associated macrophages, myeloid-derived suppressor cells, and tolerogenic dendritic cells. In this sense, cancer vaccines should not be viewed as isolated immune stimulants, but rather as interventions capable of reprogramming the tumor ecosystem when the surrounding barriers can be sufficiently overcome.

Nevertheless, the TME remains a major obstacle to durable clinical efficacy. Hypoxia, nutrient depletion, acidic metabolites, aberrant vasculature, extracellular matrix stiffness, and immunosuppressive cytokines all contribute to immune escape and can severely attenuate vaccine-driven responses. These features may prevent effector cells from reaching the tumor, reduce their viability and function once they arrive, and promote adaptive resistance after initial immune activation. Therefore, the limited success of many cancer vaccines in advanced disease likely reflects not only a lack of immunogenicity but also the inability of vaccine-induced immunity to overcome the multilayered suppressive architecture of the TME.

Taken together, the therapeutic potential of cancer vaccines will depend on strategies that integrate antigen-specific immune activation with deliberate modulation of the TME. Future success is likely to come from combination approaches that pair vaccines with checkpoint inhibitors, myeloid-targeting agents, metabolic modulators, stromal reprogramming strategies, and agents that improve vascular normalization or dendritic cell function [111,121]. Such integrated approaches may shift the balance from immune suppression toward immune activation, thereby enabling more effective, sustained, and clinically meaningful anti-tumor responses. In this framework, understanding and targeting the crosstalk between cancer vaccines and the TME is essential for advancing the next generation of rational cancer immunotherapy [193].

### 8.2. Future Perspectives

With the rapid development of high-throughput multi-omics, single-cell spatial transcriptomics, artificial intelligence-based antigen screening, and intelligent nano-delivery technologies, personalized neoantigen vaccines will become a core direction for cancer immunotherapy [5,241,243,244]. Precision timing-control strategies based on TME immunophenotyping will enable one-patient-one-regimen individualized customization of vaccine combination therapies [37,38,245,246]. Continuous iterations of novel delivery technologies such as intelligent biomimetic nanocarriers and in situ injectable delivery systems will effectively address the challenges of insufficient tissue penetration, poor targeting, and weak in vivo stability of vaccines in solid tumors [14,99,111,136,190,247]. The cross-disciplinary combination of cancer vaccines with cellular immunotherapies such as CAR-T and CAR-NK [48,211], as well as the combined use of trained immunity inducers (e.g., BCG, β-glucan) with vaccines [242,248], will build a multi-dimensional, three-dimensional anti-tumor immune network. In-depth elucidation of the molecular mechanisms governing the bidirectional crosstalk between cancer vaccines and the TME, together with continuous breakthroughs in key technologies, including antigen screening, adjuvant development, delivery carriers, and optimization of combination regimens, will open up new horizons for precision cancer immunotherapy [16,69,231,249].

## Data Availability

The data supporting this study are available from the corresponding author upon reasonable request.

## References

[1] Yoon J., Moon H., Jeon Y., Choe S., Yoon H. (2025). Signature Gene Mutations in Colorectal Cancer: Potential Neoantigens for Cancer Vaccines. Int. J. Mol. Sci..

[2] Jia W., Shen X., Guo Z., Cheng X., Zhao R. (2024). The future of cancer vaccines against colorectal cancer. Expert Opin. Biol. Ther..

[3] Rota M., Torresan I., Palmerio S., Tasselli E., Rossi A., Zivi A., Zacchi F. (2026). The role of therapeutic cancer vaccines in the modern immunotherapy era: State of the art with recent progress and future challenges. Crit. Rev. Oncol. Hematol..

[4] McMillan M.T., Soares K.C. (2025). Advances in Vaccine-Based Therapies for Pancreatic Cancer. J. Gastrointest. Cancer.

[5] Zhang J., Xiao L., Zhang Y., Jin G., Zheng K. (2025). AI-powered vaccine breakthroughs: Targeting pancreatic cancer with neoantigens and combination therapies. Biochim. Biophys. Acta Rev. Cancer.

[6] Koo S.L., Wang W.W., Toh H.C. (2018). Cancer Immunotherapy—The Target is Precisely on The Cancer and Also Not. Ann. Acad. Med. Singap..

[7] Lu Z., Zhu W., Liu X. (2025). Application prospects of tumor vaccines for pancreatic cancer: From TAAs to TSAs and combination strategies. Semin. Oncol..

[8] Saju A.F., Mukundan A., Divyashree M., Chandrashekhar R., Mahadev R.A. (2025). RNA diagnostics and therapeutics: A comprehensive review. RNA Biol..

[9] Feng H., Jin Y., Wu B. (2025). Strategies for neoantigen screening and immunogenicity validation in cancer immunotherapy (Review). Int. J. Oncol..

[10] Baddal B. (2019). Next-generation technologies for studying host-pathogen interactions: A focus on dual transcriptomics, CRISPR/Cas9 screening and organs-on-chips. Pathog. Dis..

[11] Noe A., Cargill T.N., Nielsen C.M., Russell A.J.C., Barnes E. (2020). The Application of Single-Cell RNA Sequencing in Vaccinology. J. Immunol. Res..

[12] Tang Z., Zha L., Liang R., Li T. (2026). Lung cancer vaccines to enhance immune checkpoint inhibitor therapy: Evidence and future perspectives. J. Hematol. Oncol..

[13] Adusumilli P.S., Cha E., Cornfeld M., Davis T., Diab A., Dubensky T.W., Evans E., Grogan J.L., Irving B.A., Leidner R.S. (2017). New Cancer Immunotherapy Agents in Development: A report from an associated program of the 31(st)Annual Meeting of the Society for Immunotherapy of Cancer, 2016. J. Immunother. Cancer.

[14] Liu N., Wang X., Wang Z., Kan Y., Fang Y., Gao J., Kong X., Wang J. (2025). Nanomaterials-driven in situ vaccination: A novel frontier in tumor immunotherapy. J. Hematol. Oncol..

[15] Weber J.S., Carlino M.S., Khattak A., Meniawy T., Ansstas G., Taylor M.H., Kim K.B., McKean M., Long G.V., Sullivan R.J. (2024). Individualised neoantigen therapy mRNA-4157 (V940) plus pembrolizumab versus pembrolizumab monotherapy in resected melanoma (KEYNOTE-942): A randomised, phase 2b study. Lancet.

[16] Farhangnia P., Khorramdelazad H., Nickho H., Delbandi A.A. (2024). Current and future immunotherapeutic approaches in pancreatic cancer treatment. J. Hematol. Oncol..

[17] Rojas L.A., Sethna Z., Soares K.C., Olcese C., Pang N., Patterson E., Lihm J., Ceglia N., Guasp P., Chu A. (2023). Personalized RNA neoantigen vaccines stimulate T cells in pancreatic cancer. Nature.

[18] Palmer C.D., Rappaport A.R., Davis M.J., Hart M.G., Scallan C.D., Hong S.J., Gitlin L., Kraemer L.D., Kounlavouth S., Yang A. (2022). Individualized, heterologous chimpanzee adenovirus and self-amplifying mRNA neoantigen vaccine for advanced metastatic solid tumors: Phase 1 trial interim results. Nat. Med..

[19] Awad M.M., Govindan R., Balogh K.N., Spigel D.R., Garon E.B., Bushway M.E., Poran A., Sheen J.H., Kohler V., Esaulova E. (2022). Personalized neoantigen vaccine NEO-PV-01 with chemotherapy and anti-PD-1 as first-line treatment for non-squamous non-small cell lung cancer. Cancer Cell.

[20] Sun C., Nagaoka K., Kobayashi Y., Nakagawa H., Kakimi K., Nakajima J. (2021). Neoantigen Dendritic Cell Vaccination Combined with Anti-CD38 and CpG Elicits Anti-Tumor Immunity against the Immune Checkpoint Therapy-Resistant Murine Lung Cancer Cell Line LLC1. Cancers.

[21] Lee K.W., Yam J.W.P., Mao X. (2023). Dendritic Cell Vaccines: A Shift from Conventional Approach to New Generations. Cells.

[22] Tseha S.T. (2022). Role of Adenoviruses in Cancer Therapy. Front. Oncol..

[23] Gruenerbel L., Heinrich F., Bohlhoff-Martin J., Roper L., Machens H.G., Gruenerbel A., Schillinger M., Kist A., Wenninger F., Richter M. (2023). Wearable Prophylaxis Tool for AI-Driven Identification of Early Warning Patterns of Pressure Ulcers. Bioengineering.

[24] Wang X., Niu Y., Bian F. (2025). The progress of tumor vaccines clinical trials in non-small cell lung cancer. Clin. Transl. Oncol..

[25] Niu B., Xu J., Zhang L. (2026). Tumor Vaccines in Hepatocellular Carcinoma: Advances, Challenges, and the Path Toward Precision Immunotherapy. J. Clin. Transl. Hepatol..

[26] Pandya A., Shah Y., Kothari N., Postwala H., Shah A., Parekh P., Chorawala M.R. (2023). The future of cancer immunotherapy: DNA vaccines leading the way. Med. Oncol..

[27] Kumar A., Dixit S., Srinivasan K.M.D., Vincent P. (2024). Personalized cancer vaccine design using AI-powered technologies. Front. Immunol..

[28] Magoola M., Niazi S.K. (2025). Engineering Universal Cancer Immunity: Non-Tumor-Specific mRNA Vaccines Trigger Epitope Spreading in Cold Tumors. Vaccines.

[29] Moseman J.E., Shim D., Jeon D., Rastogi I., Schneider K.M., McNeel D.G. (2025). Messenger RNA and Plasmid DNA Vaccines for the Treatment of Cancer. Vaccines.

[30] Wu J., Cai J. (2021). Dilemma and Challenge of Immunotherapy for Pancreatic Cancer. Dig. Dis. Sci..

[31] Babar Q., Saeed A., Tabish T.A., Sarwar M., Thorat N.D. (2023). Targeting the tumor microenvironment: Potential strategy for cancer therapeutics. Biochim. Biophys. Acta Mol. Basis Dis..

[32] Nair S.S., Weil R., Dovey Z., Davis A., Tewari A.K. (2020). The Tumor Microenvironment and Immunotherapy in Prostate and Bladder Cancer. Urol. Clin. N. Am..

[33] Jiang J., Mei J., Yi S., Feng C., Ma Y., Liu Y., Liu Y., Chen C. (2022). Tumor associated macrophage and microbe: The potential targets of tumor vaccine delivery. Adv. Drug Deliv. Rev..

[34] Liu Y., Li S., Chen L., Lin L., Xu C., Qiu H., Li X., Cao H., Liu K. (2024). Global trends in tumor microenvironment-related research on tumor vaccine: A review and bibliometric analysis. Front. Immunol..

[35] Yu J., Kong X., Feng Y. (2025). Tumor microenvironment-driven resistance to immunotherapy in non-small cell lung cancer: Strategies for Cold-to-Hot tumor transformation. Cancer Drug Resist..

[36] Bonaventura P., Shekarian T., Alcazer V., Valladeau-Guilemond J., Valsesia-Wittmann S., Amigorena S., Caux C., Depil S. (2019). Cold Tumors: A Therapeutic Challenge for Immunotherapy. Front. Immunol..

[37] Wang M., Wang S., Desai J., Trapani J.A., Neeson P.J. (2020). Therapeutic strategies to remodel immunologically cold tumors. Clin. Transl. Immunol..

[38] Galon J., Bruni D. (2019). Approaches to treat immune hot, altered and cold tumours with combination immunotherapies. Nat. Rev. Drug Discov..

[39] Yang J., Zhang C. (2020). Regulation of cancer-immunity cycle and tumor microenvironment by nanobiomaterials to enhance tumor immunotherapy. Wiley Interdiscip. Rev. Nanomed. Nanobiotechnol..

[40] Pan Y., Song X., Wang Y., Wei J. (2021). Firing up the Tumor Microenvironment with Nanoparticle-Based Therapies. Pharmaceutics.

[41] Tomaszewski W., Sanchez-Perez L., Gajewski T.F., Sampson J.H. (2019). Brain Tumor Microenvironment and Host State: Implications for Immunotherapy. Clin. Cancer Res..

[42] Liu J., Zhang B., Huang B., Zhang K., Guo F., Wang Z., Shang D. (2024). A stumbling block in pancreatic cancer treatment: Drug resistance signaling networks. Front. Cell Dev. Biol..

[43] Imani S., Farghadani R., Roozitalab G., Maghsoudloo M., Emadi M., Moradi A., Abedi B., Jabbarzadeh Kaboli P. (2025). Reprogramming the breast tumor immune microenvironment: Cold-to-hot transition for enhanced immunotherapy. J. Exp. Clin. Cancer Res..

[44] Runcie K.D., Dallos M.C. (2021). Prostate Cancer Immunotherapy—Finally in From the Cold?. Curr. Oncol. Rep..

[45] Xu P., Wasielewski L.J., Yang J.C., Cai D., Evans C.P., Murphy W.J., Liu C. (2022). The Immunotherapy and Immunosuppressive Signaling in Therapy-Resistant Prostate Cancer. Biomedicines.

[46] Maia M.C., Hansen A.R. (2017). A comprehensive review of immunotherapies in prostate cancer. Crit. Rev. Oncol. Hematol..

[47] Liu D., Liu L., Zhao X., Zhang X., Chen X., Che X., Wu G. (2025). A comprehensive review on targeting diverse immune cells for anticancer therapy: Beyond immune checkpoint inhibitors. Crit. Rev. Oncol. Hematol..

[48] Alqarni A., Jasim S.A., Altalbawy F.M.A., Kaur H., Kaur I., Rodriguez-Benites C., Deorari M., Alwaily E.R., Al-Ani A.M., Redhee A.H. (2024). Challenges and opportunities for cancer stem cell-targeted immunotherapies include immune checkpoint inhibitor, cancer stem cell-dendritic cell vaccine, chimeric antigen receptor immune cells, and modified exosomes. J. Biochem. Mol. Toxicol..

[49] Lorizio D., Silginer M., Friesen J., Epstein A.L., Weller M., Roth P. (2025). Simultaneous TGF-β and GITR pathway modulation promotes anti-tumor immunity in glioma. Cancer Immunol. Immunother..

[50] Moreau J.M., Velegraki M., Bolyard C., Rosenblum M.D., Li Z. (2022). Transforming growth factor-β1 in regulatory T cell biology. Sci. Immunol..

[51] Song J., Lin Z., Liu Q., Huang S., Han L., Fang Y., Zhong P., Dou R., Xiang Z., Zheng J. (2022). MiR-192-5p/RB1/NF-kappaBp65 signaling axis promotes IL-10 secretion during gastric cancer EMT to induce Treg cell differentiation in the tumour microenvironment. Clin. Transl. Med..

[52] Amer H., Flanagan K.L., Kampan N.C., Itsiopoulos C., Scott C.L., Kartikasari A.E.R., Plebanski M. (2025). Interleukin-6 Is a Crucial Factor in Shaping the Inflammatory Tumor Microenvironment in Ovarian Cancer and Determining Its Hot or Cold Nature with Diagnostic and Prognostic Utilities. Cancers.

[53] Naik A., Decock J. (2020). Lactate Metabolism and Immune Modulation in Breast Cancer: A Focused Review on Triple Negative Breast Tumors. Front. Oncol..

[54] Lv B., Wang Y., Ma D., Cheng W., Liu J., Yong T., Chen H., Wang C. (2022). Immunotherapy: Reshape the Tumor Immune Microenvironment. Front. Immunol..

[55] Brand A., Singer K., Koehl G.E., Kolitzus M., Schoenhammer G., Thiel A., Matos C., Bruss C., Klobuch S., Peter K. (2016). LDHA-Associated Lactic Acid Production Blunts Tumor Immunosurveillance by T and NK Cells. Cell Metab..

[56] Fischer K., Hoffmann P., Voelkl S., Meidenbauer N., Ammer J., Edinger M., Gottfried E., Schwarz S., Rothe G., Hoves S. (2007). Inhibitory effect of tumor cell-derived lactic acid on human T cells. Blood.

[57] Rodriguez-Pampin I., Gonzalez-Pico L., Selas A., Andujar A., Prieto-Diaz R., Sotelo E. (2025). Targeting the adenosinergic axis in cancer immunotherapy: Insights into A(2A) and A(2B) receptors and novel clinical combination strategies. Pharmacol. Rev..

[58] Carlsen L., Zhang S., Tian X., De La Cruz A., George A., Arnoff T.E., El-Deiry W.S. (2023). The role of p53 in anti-tumor immunity and response to immunotherapy. Front. Mol. Biosci..

[59] Gallus M., Kwok D., Lakshmanachetty S., Yamamichi A., Okada H. (2023). Immunotherapy Approaches in Isocitrate-Dehydrogenase-Mutant Low-Grade Glioma. Cancers.

[60] Chen J., Duan Y., Che J., Zhu J. (2024). Dysfunction of dendritic cells in tumor microenvironment and immunotherapy. Cancer Commun..

[61] Gupta Y.H., Khanom A., Acton S.E. (2022). Control of Dendritic Cell Function Within the Tumour Microenvironment. Front. Immunol..

[62] Zhao X., Liu B., William W.N., Tsanov K.M., Ho Y.J., Barriga F.M., Lim R.J., Trifas M., Khandekar A., Du Y. (2025). Interferon Epsilon Loss Is Elusive 9p21 Link to Immune-Cold Tumors, Resistant to Immune Checkpoint Therapy, and Endogenous CXCL9/10 Induction. J. Thorac. Oncol..

[63] Lin P., Lin Y., Chen X., Zhao X., Cui L. (2025). Decoding MHC loss: Molecular mechanisms and implications for immune resistance in cancer. Clin. Transl. Med..

[64] Yang Z., Liu K., Li H., Li Y., Sun Q., Shang W., Wang M., Yang Y., Liu H., Yin D. (2025). Oncolytic Hydrogel Enhances Immune Checkpoint Blockade by Generating In Situ Vaccine, Remodeling Tumor Physical and Metabolic Barriers. ACS Nano.

[65] Kwon W.A., Joung J.Y. (2025). Immunotherapy in Prostate Cancer: From a “Cold” Tumor to a “Hot” Prospect. Cancers.

[66] Kane G.I., Lusi C.F., Brassil M.L., Atukorale P.U. (2024). Engineering approaches for innate immune-mediated tumor microenvironment remodeling. Immunooncol Technol..

[67] Li Y.R., Wilson M., Yang L. (2022). Target tumor microenvironment by innate T cells. Front. Immunol..

[68] Li C., Yu X., Han X., Lian C., Wang Z., Shao S., Shao F., Wang H., Ma S., Liu J. (2024). Innate immune cells in tumor microenvironment: A new frontier in cancer immunotherapy. iScience.

[69] Wang Y., Jiang C., Zhou H., Han R. (2025). Transforming cancer immunotherapy: Integration of distinct immune-based approaches as redefined dual immunotherapy with potential third-sensitizer. Exp. Hematol. Oncol..

[70] Khleif S.N., Gupta S. (2025). Cancer vaccines as enablers of immunotherapy. Nat. Immunol..

[71] Zhang H., Li S., Liu S., Liao Y., Liu H., Yang M., Chen P. (2025). Therapeutic targeting of cell death-immune crosstalk in cancer to rewire the tumor immune microenvironment. Mol. Cancer..

[72] Wang R., Wu J., Lin Y., Xiao Y., Yang B., Yao S., Pan T., Fu Z., Li S., Wang C. (2025). An epitope-directed mRNA vaccine inhibits tumor metastasis through the blockade of MICA/B α1/2 shedding. Cell Rep. Med..

[73] Chen Z., Qin Y.T., Li Q.R., He J.L., Deng X.C., Zhang Y., Yang H.D., Feng J., Sun Y.X., Zhang X.Z. (2025). Layer-by-Layer Deposition of Antigen Peptides on *Bifidobacterium* for Subintestinal Lymphatic System-Guided Personalized Tumor Immunotherapy. Adv. Mater..

[74] Zhao T., Cai Y., Jiang Y., He X., Wei Y., Yu Y., Tian X. (2023). Vaccine adjuvants: Mechanisms and platforms. Signal Transduct. Target. Ther..

[75] Liang S., Gao S., Fu S., Yuan S., Liu J., Liang M., Han L., Zhang Z., Liu Y., Zhang N. (2025). Screening Natural Cholesterol Analogs to Assemble Self-Adjuvant Lipid Nanoparticles for Antigens Tagging Guided Therapeutic Tumor Vaccine. Adv. Mater..

[76] Wang Y., Song W., Xu Q., Liu Y., Liu H., Guo R., Chiou C.J., Gao K., Jin B., Chen C. (2023). Adjuvant DNA vaccine pNMM promotes enhanced specific immunity and anti-tumor effects. Hum. Vaccines Immunother..

[77] Latha T.S., Panati K., Gowd D.S., Reddy M.C., Lomada D. (2014). Ovarian cancer biology and immunotherapy. Int. Rev. Immunol..

[78] Li G., Srinivasan S., Wang L., Ma C., Guo K., Xiao W., Liao W., Mishra S., Zhang X., Qiu Y. (2022). TGF-β-dependent lymphoid tissue residency of stem-like T cells limits response to tumor vaccine. Nat. Commun..

[79] Chrisikos T.T., Zhou Y., Li H.S., Babcock R.L., Wan X., Patel B., Newton K., Mancuso J.J., Watowich S.S. (2020). STAT3 Inhibits CD103(+) cDC1 Vaccine Efficacy in Murine Breast Cancer. Cancers.

[80] Chekaoui A., Ertl H.C.J. (2021). PPARα Agonist Fenofibrate Enhances Cancer Vaccine Efficacy. Cancer Res..

[81] Yao Y., Luo Y., Wang Z., Yin E., Liu C., Zheng S., Wang X., Tang X., Sun N., He J. (2025). Tumor-secreted AGR2 induces dendritic cell dysfunction and impairs immunotherapy efficacy in LKB1-deficient cancer. J. Immunother. Cancer..

[82] Jiang J., Zhou H., Ni C., Hu X., Mou Y., Huang D., Yang L. (2019). Immunotherapy in pancreatic cancer: New hope or mission impossible?. Cancer Lett..

[83] Hu Y., Nie W., Lyu L., Zhang X., Wang W., Zhang Y., He S., Guo A., Liu F., Wang B. (2024). Tumor-Microenvironment-Activatable Nanoparticle Mediating Immunogene Therapy and M2 Macrophage-Targeted Inhibitor for Synergistic Cancer Immunotherapy. ACS Nano..

[84] Niedbala M., Malarz K., Sharma G., Kramer-Marek G., Kaspera W. (2022). Glioblastoma: Pitfalls and Opportunities of Immunotherapeutic Combinations. OncoTargets Ther..

[85] Sato R., Yamaki H., Komatsuda H., Wakisaka R., Inoue T., Kumai T., Takahara M. (2024). Exploring Immunological Effects and Novel Immune Adjuvants in Immunotherapy for Salivary Gland Cancers. Cancers.

[86] Shibata H., Kuroki M., Kawaura R., Yamada T., Iinuma R., Ishihara H., Okuda H., Mori K., Ogawa T. (2025). Head and Neck Cancer Immunotherapy: Overcoming Limitations and Enhancing Efficacy. Cancer Sci..

[87] Yang C., Xia B.R., Zhang Z.C., Zhang Y.J., Lou G., Jin W.L. (2020). Immunotherapy for Ovarian Cancer: Adjuvant, Combination, and Neoadjuvant. Front. Immunol..

[88] Song R., Li M., Tang S., Zhang G. (2026). Immunotherapy for virus-related hepatocellular carcinoma: Recent progress and future directions. Ann. Med..

[89] El Meligy O.A., Elemam N.M., Hassan W.A., Talaat I.M. (2025). Tumor-Immune Interactions in Pediatric Oral Rhabdomyosarcoma: A Narrative Review on Immuno-Oncology and Emerging Therapies. Children.

[90] Plant-Fox A.S., O’Halloran K., Goldman S. (2021). Pediatric brain tumors: The era of molecular diagnostics, targeted and immune-based therapeutics, and a focus on long term neurologic sequelae. Curr. Probl. Cancer.

[91] Wang Y., Li G., Su J., Liu Y., Zhang X., Zhang G., Wu Z., Li J., Zhang Y., Wang X. (2024). Spatiotemporal Controllable Sono-Nanovaccines Driven by Free-Field Based Whole-Body Ultrasound for Personalized Cancer Therapy. Adv. Sci..

[92] Xu X., Zheng J., Liang N., Zhang X., Shabiti S., Wang Z., Yu S., Pan Z.Y., Li W., Cai L. (2024). Bioorthogonal/Ultrasound Activated Oncolytic Pyroptosis Amplifies In Situ Tumor Vaccination for Boosting Antitumor Immunity. ACS Nano.

[93] Duan X., Chan C., Lin W. (2019). Nanoparticle-Mediated Immunogenic Cell Death Enables and Potentiates Cancer Immunotherapy. Angew. Chem. Int. Ed. Engl..

[94] Iwahori K. (2020). Cytotoxic CD8(+) Lymphocytes in the Tumor Microenvironment. Adv. Exp. Med. Biol..

[95] Montauti E., Oh D.Y., Fong L. (2024). CD4(+) T cells in antitumor immunity. Trends Cancer.

[96] Jin M.Z., Wang X.P. (2021). Immunogenic Cell Death-Based Cancer Vaccines. Front. Immunol..

[97] Cecil D., Park K.H., Curtis B., Corulli L., Disis M.N. (2021). Type I T cells sensitize treatment refractory tumors to chemotherapy through inhibition of oncogenic signaling pathways. J. Immunother. Cancer..

[98] Tokunaga R., Zhang W., Naseem M., Puccini A., Berger M.D., Soni S., McSkane M., Baba H., Lenz H.J. (2018). CXCL9, CXCL10, CXCL11/CXCR3 axis for immune activation—A target for novel cancer therapy. Cancer Treat. Rev..

[99] Thiruppathi J., Vijayan V., Park I.K., Lee S.E., Rhee J.H. (2024). Enhancing cancer immunotherapy with photodynamic therapy and nanoparticle: Making tumor microenvironment hotter to make immunotherapeutic work better. Front. Immunol..

[100] Rameshbabu S., Labadie B.W., Argulian A., Patnaik A. (2021). Targeting Innate Immunity in Cancer Therapy. Vaccines.

[101] Jeon D., Hill E., McNeel D.G. (2024). Toll-like receptor agonists as cancer vaccine adjuvants. Hum. Vaccines Immunother..

[102] Wang X., Huang Z., Xing L., Shang L., Jiang J., Deng C., Yu W., Peng L., Yang H., Zheng X. (2025). STING agonist-based ER-targeting molecules boost antigen cross-presentation. Nature.

[103] Chen Y., Du Z., Jiang W., Kong L., Huang D., Zeng C., Liu H. (2025). Engineered IFNγ-loaded exosomes reprogram macrophage polarization and suppress tumor cell proliferation in vitro. Int. Immunopharmacol..

[104] Zhang Y., Lei Y., Ou Q., Chen M., Tian S., Tang J., Li R., Liang Q., Chen Z., Wang C. (2024). Listeria-vectored cervical cancer vaccine candidate strains reduce MDSCs via the JAK-STAT signaling pathway. BMC Biol..

[105] Wang X., Xue S., Li Y., Yang M., Yu H., Wang M., Li S., Lu Z., Luo M. (2026). CD300ld blockade overcomes PMN-MDSC-mediated vaccine resistance in advanced tumors. J. Immunother. Cancer.

[106] Tadic S., Garcia-Sanmartin J., Narro-Iniguez J., Martinez A. (2025). A Stable RNA Vaccine Against the Regulatory Peptide Adrenomedullin Reduces Angiogenesis and Tumor Burden in a Subcutaneous Melanoma Model Without Inducing an Immunosuppressive Tumor Microenvironment. Int. J. Mol. Sci..

[107] Wagner S.C., Ichim T.E., Bogin V., Min W.P., Silva F., Patel A.N., Kesari S. (2017). Induction and characterization of anti-tumor endothelium immunity elicited by ValloVax therapeutic cancer vaccine. Oncotarget.

[108] Osborne N., Sundseth R., Gay M.D., Cao H., Tucker R.D., Nadella S., Wang S., Liu X., Kroemer A., Sutton L. (2019). Vaccine against gastrin, a polyclonal antibody stimulator, decreases pancreatic cancer metastases. Am. J. Physiol. Gastrointest. Liver Physiol..

[109] Perez-Penco M., Lara de la Torre L., Lecoq I., Martinenaite E., Andersen M.H. (2024). TGFβ-specific T cells induced by a TGFβ-derived immune modulatory vaccine both directly and indirectly modulate the phenotype of tumor-associated macrophages and fibroblasts. J. Immunother. Cancer.

[110] Ring S.S., Cupovic J., Onder L., Lutge M., Perez-Shibayama C., Gil-Cruz C., Scandella E., De Martin A., Mörbe U., Hartmann F. (2021). Viral vector-mediated reprogramming of the fibroblastic tumor stroma sustains curative melanoma treatment. Nat. Commun..

[111] Ahmad I., Altameemi K.K.A., Hani M.M., Ali A.M., Shareef H.K., Hassan Z.F., Alubiady M.H.S., Al-Abdeen S.H.Z., Shakier H.G., Redhee A.H. (2025). Shifting cold to hot tumors by nanoparticle-loaded drugs and products. Clin. Transl. Oncol..

[112] O’Connell B.C., Hubbard C., Zizlsperger N., Fitzgerald D., Kutok J.L., Varner J., Ilaria R., Cobleigh M.A., Juric D., Tkaczuk K.H.R. (2024). Eganelisib combined with immune checkpoint inhibitor therapy and chemotherapy in frontline metastatic triple-negative breast cancer triggers macrophage reprogramming, immune activation and extracellular matrix reorganization in the tumor microenvironment. J. Immunother. Cancer..

[113] Lee C., Lee J., Jeong M., Nam D., Rhee I. (2025). Emerging strategies for targeting tumor-associated macrophages in glioblastoma: A focus on chemotaxis blockade. Life Sci..

[114] Yang L., Zhang Y. (2017). Tumor-associated macrophages: From basic research to clinical application. J. Hematol. Oncol..

[115] Knorr D.A., Blanchard L., Leidner R.S., Jensen S.M., Meng R., Jones A., Ballesteros-Merino C., Bell R.B., Baez M., Marino A. (2024). FcγRIIB Is an Immune Checkpoint Limiting the Activity of Treg-Targeting Antibodies in the Tumor Microenvironment. Cancer Immunol. Res..

[116] Fattori S., Le Roy A., Houacine J., Robert L., Abes R., Gorvel L., Granjeaud S., Rouvière M.S., Ben Amara A., Boucherit N. (2023). CD25high Effector Regulatory T Cells Hamper Responses to PD-1 Blockade in Triple-Negative Breast Cancer. Cancer Res..

[117] Kwong T.T., Xiong Z., Zhang Y., Wu H., Cao J., Pak-Chun Wong P., Liu X., Wang J., Wong C.H., Man-Kit Tse G. (2025). Overcoming immunotherapy resistance in hepatocellular carcinoma by targeting myeloid IL-8/CXCR2 signaling. Mol. Ther..

[118] Deshpande N.U., Bianchi A., Amirian H., De Castro Silva I., Rafie C.I., Surnar B., Rajkumar K., Ashokan A., Ogobuiro I.C., Patel M. (2025). Cell-specific nanoengineering strategy to disrupt tolerogenic signaling from myeloid-derived suppressor cells and invigorate antitumor immunity in pancreatic cancer. Cancer Immunol. Immunother..

[119] Ao J.Y., Zhu X.D., Chai Z.T., Cai H., Zhang Y.Y., Zhang K.Z., Kong L.Q., Zhang N., Ye B.G., Ma D.N. (2017). Colony-Stimulating Factor 1 Receptor Blockade Inhibits Tumor Growth by Altering the Polarization of Tumor-Associated Macrophages in Hepatocellular Carcinoma. Mol. Cancer Ther..

[120] Valero J.G., Matas-Cespedes A., Arenas F., Rodriguez V., Carreras J., Serrat N., Guerrero-Hernández M., Yahiaoui A., Balagué O., Martin S. (2021). The receptor of the colony-stimulating factor-1 (CSF-1R) is a novel prognostic factor and therapeutic target in follicular lymphoma. Leukemia.

[121] Sridaran D., Bradshaw E., DeSelm C., Pachynski R., Mahajan K., Mahajan N.P. (2023). Prostate cancer immunotherapy: Improving clinical outcomes with a multi-pronged approach. Cell Rep. Med..

[122] Maldonado M.D.M., Schlom J., Hamilton D.H. (2023). Blockade of tumor-derived colony-stimulating factor 1 (CSF1) promotes an immune-permissive tumor microenvironment. Cancer Immunol. Immunother..

[123] Borodovsky A., Barbon C.M., Wang Y., Ye M., Prickett L., Chandra D., Shaw J., Deng N., Sachsenmeier K., Clarke J.D. (2020). Small molecule AZD4635 inhibitor of A(2A)R signaling rescues immune cell function including CD103(+) dendritic cells enhancing anti-tumor immunity. J. Immunother. Cancer..

[124] Yang L., Zhang Y., Yang L. (2024). Adenosine signaling in tumor-associated macrophages and targeting adenosine signaling for cancer therapy. Cancer Biol. Med..

[125] Blair A.B., Wang J., Davelaar J., Baker A., Li K., Niu N., Wang J., Shao Y., Funes V., Li P. (2022). Dual Stromal Targeting Sensitizes Pancreatic Adenocarcinoma for Anti-Programmed Cell Death Protein 1 Therapy. Gastroenterology.

[126] Blair A.B., Kim V.M., Muth S.T., Saung M.T., Lokker N., Blouw B., Armstrong T.D., Jaffee E.M., Tsujikawa T., Coussens L.M. (2019). Dissecting the Stromal Signaling and Regulation of Myeloid Cells and Memory Effector T Cells in Pancreatic Cancer. Clin. Cancer Res..

[127] Safaei S., Sajed R., Shariftabrizi A., Dorafshan S., Saeednejad Zanjani L., Dehghan Manshadi M., Madjd Z., Ghods R. (2023). Tumor matrix stiffness provides fertile soil for cancer stem cells. Cancer Cell Int..

[128] Lorusso G., Wyss C.B., Kuonen F., Vannini N., Billottet C., Duffey N., Pineau R., Lan Q., Wirapati P., Barras D. (2022). Connexins orchestrate progression of breast cancer metastasis to the brain by promoting FAK activation. Sci. Transl. Med..

[129] Yu D., Zuo Y., Qi M., Zhong H. (2025). Advances in the combination of immunoadjuvants and tumor ablation: Mediating immune effects to enhance anti-tumor efficacy. Biochim. Biophys. Acta Rev. Cancer.

[130] Brem S. (2024). Vagus nerve stimulation: Novel concept for the treatment of glioblastoma and solid cancers by cytokine (interleukin-6) reduction, attenuating the SASP, enhancing tumor immunity. Brain Behav. Immun. Health.

[131] Gerard C.L., Delyon J., Wicky A., Homicsko K., Cuendet M.A., Michielin O. (2021). Turning tumors from cold to inflamed to improve immunotherapy response. Cancer Treat. Rev..

[132] Zheng S., Wang W., Shen L., Yao Y., Xia W., Ni C. (2024). Tumor battlefield within inflamed, excluded or desert immune phenotypes: The mechanisms and strategies. Exp. Hematol. Oncol..

[133] Hogg G.D., Weinstein A.G., Kingston N.L., Liu X., Dres O.M., Kang L.I., Lander V.E., Kao Y.L., Ahmad F., Knolhoff B.L. (2025). Combined Flt3L and CD40 agonism restores dendritic cell-driven T cell immunity in pancreatic cancer. Sci. Immunol..

[134] Tan Y.S., Sansanaphongpricha K., Prince M.E.P., Sun D., Wolf G.T., Lei Y.L. (2018). Engineering Vaccines to Reprogram Immunity against Head and Neck Cancer. J. Dent. Res..

[135] Tran D.T., Nguyen B.Q.T., Nguyen H.T., Pham T.M.Q., Nguyen D.V.L., Nguyen T.N., Ho T.K.C., Nguyen V., Nguyen P.T.D., Tran D.H. (2026). Identification of public neoantigens with broad HLA class I coverage as candidates for off-the-shelf cancer vaccine development in colorectal cancer. Front. Immunol..

[136] Jiang Q., Xie M., Chen R., Yan F., Ye C., Li Q., Xu S., Wu W., Jia Y., Shen P. (2022). Cancer cell membrane-wrapped nanoparticles for cancer immunotherapy: A review of current developments. Front. Immunol..

[137] Zhao Z., Liu H., Li M., Zheng Y., Qi D., Chen C., Wang Z., Fu L., Han S., Yang X. (2026). Thermosensitive Hydrogel In Situ Vaccine for Lymph Node Targeting and Enhanced Immunotherapy. Adv. Healthc. Mater..

[138] Zhang Z., Xu C., Gong N., Qing G., Zhang Y., Shi Y., Brenner J.S., Li F., Xu F.J., Liang X.J. (2025). An antigen-capturing and lymph node-targeting nanoparticle for cancer immunotherapy. J. Control. Release.

[139] Li X., Yang F., Wang M., Huang X., Zeng X., Zhou L., Peng S., Zhang J. (2025). Unleashing the power of peptides in prostate cancer immunotherapy: Mechanism, facts and perspectives. Front. Pharmacol..

[140] Castro Eiro M.D., Hioki K., Li L., Wilmsen M.E.P., Kiernan C.H., Brouwers-Haspels I., van Meurs M., Zhao M., de Wit H., Grashof D.G.B. (2024). TLR9 plus STING Agonist Adjuvant Combination Induces Potent Neopeptide T Cell Immunity and Improves Immune Checkpoint Blockade Efficacy in a Tumor Model. J. Immunol..

[141] Mai J., Li Z., Xia X., Zhang J., Li J., Liu H., Shen J., Ramirez M., Li F., Li Z. (2021). Synergistic Activation of Antitumor Immunity by a Particulate Therapeutic Vaccine. Adv. Sci..

[142] Sun Y., Liu L., He H., Cui G., Zheng Y., Ye C., Qu L., Sun Y., Ji J., Lammers T. (2025). Co-activating STING-TLR9 pathways promotes radiotherapy-induced cancer vaccination. J. Control Release.

[143] Keskin D.B., Anandappa A.J., Sun J., Tirosh I., Mathewson N.D., Li S., Oliveira G., Giobbie-Hurder A., Felt K., Gjini E. (2019). Neoantigen vaccine generates intratumoral T cell responses in phase Ib glioblastoma trial. Nature.

[144] Appleton E., Hassan J., Chan Wah Hak C., Sivamanoharan N., Wilkins A., Samson A., Ono M., Harrington K.J., Melcher A., Wennerberg E. (2021). Kickstarting Immunity in Cold Tumours: Localised Tumour Therapy Combinations with Immune Checkpoint Blockade. Front. Immunol..

[145] Collins J.M., Redman J.M., Gulley J.L. (2018). Combining vaccines and immune checkpoint inhibitors to prime, expand, and facilitate effective tumor immunotherapy. Expert Rev. Vaccines.

[146] Kon E., Benhar I. (2019). Immune checkpoint inhibitor combinations: Current efforts and important aspects for success. Drug Resist. Update.

[147] Mooradian M.J., Sullivan R.J. (2017). Immunomodulatory effects of current cancer treatment and the consequences for follow-up immunotherapeutics. Future Oncol..

[148] Pieper A.A., Zangl L.M., Speigelman D.V., Feils A.S., Hoefges A., Jagodinsky J.C., Felder M.A., Tsarovsky N.W., Arthur I.S., Brown R.J. (2021). Radiation Augments the Local Anti-Tumor Effect of In Situ Vaccine with CpG-Oligodeoxynucleotides and Anti-OX40 in Immunologically Cold Tumor Models. Front. Immunol..

[149] Vreeland T.J., Clifton G.T., Herbert G.S., Hale D.F., Jackson D.O., Berry J.S., Peoples G.E. (2016). Gaining ground on a cure through synergy: Combining checkpoint inhibitors with cancer vaccines. Expert Rev. Clin. Immunol..

[150] Wilky B.A. (2019). Immune checkpoint inhibitors: The linchpins of modern immunotherapy. Immunol. Rev..

[151] Bansal D., Reimers M.A., Knoche E.M., Pachynski R.K. (2021). Immunotherapy and Immunotherapy Combinations in Metastatic Castration-Resistant Prostate Cancer. Cancers.

[152] Chesney J.A., Puzanov I., Collichio F.A., Singh P., Milhem M.M., Glaspy J., Hamid O., Ross M., Friedlander P., Garbe C. (2023). Talimogene laherparepvec in combination with ipilimumab versus ipilimumab alone for advanced melanoma: 5-year final analysis of a multicenter, randomized, open-label, phase II trial. J. Immunother. Cancer..

[153] Antonia S.J., Villegas A., Daniel D., Vicente D., Murakami S., Hui R., Yokoi T., Chiappori A., Lee K.H., de Wit M. (2017). Durvalumab after Chemoradiotherapy in Stage III Non-Small-Cell Lung Cancer. N. Engl. J. Med..

[154] Finn R.S., Qin S., Ikeda M., Galle P.R., Ducreux M., Kim T.Y., Kudo M., Breder V., Merle P., Kaseb A.O. (2020). Atezolizumab plus Bevacizumab in Unresectable Hepatocellular Carcinoma. N. Engl. J. Med..

[155] Abou-Alfa G.K., Lau G., Kudo M., Chan S.L., Kelley R.K., Furuse J., Sukeepaisarnjaroen W., Kang Y.K., Van Dao T., De Toni E.N. (2022). Tremelimumab plus Durvalumab in Unresectable Hepatocellular Carcinoma. NEJM Evid..

[156] Rimassa L., Chan S.L., Sangro B., Lau G., Kudo M., Reig M., Breder V., Ryu M.H., Ostapenko Y., Sukeepaisarnjaroen W. (2025). Five-year overall survival update from the HIMALAYA study of tremelimumab plus durvalumab in unresectable HCC. J. Hepatol..

[157] Lau G., Abou-Alfa G.K., Cheng A.L., Sukeepaisarnjaroen W., Van Dao T., Kang Y.K., Thungappa S.C., Kudo M., Sangro B., Kelley R.K. (2025). Outcomes in the Asian subgroup of the phase III randomised HIMALAYA study of tremelimumab plus durvalumab in unresectable hepatocellular carcinoma. J. Hepatol..

[158] Kwok T., Silva-Junior I.A., Korpe S., Dong H., Lancaster J.N. (2025). Macrophage repolarization by immune checkpoint blockade drives T cell engagement in the tumor microenvironment. iScience.

[159] Qiu F., Sun Y., Lim D.W. (2026). Therapeutic vaccines targeting solid tumors: A series of clinical trial analysis over the past decade. Transl. Oncol..

[160] Deng D., Li G., Xia X., Xu S., Gao L., Zhang L., Yao W., Tian H., Gao X. (2024). Nitrated T cell epitope linked vaccine targeting CD47 elicits antitumor immune responses and acts synergistically with vaccine targeting PDL1. Int. Immunopharmacol..

[161] Chu F., Hou P., Zhu H., Gao Y., Wang X., He W., Ren J., Li M., Liu Y., He D.C. (2024). PBLD enhances antiviral innate immunity by promoting the p53-USP4-MAVS signaling axis. Proc. Natl. Acad. Sci. USA..

[162] Taylor J.L., Kokolus K.M., Basse P.H., Filderman J.N., Cosgrove C.E., Watkins S.C., Gambotto A., Lowe D.B., Edwards R.P., Kalinski P. (2024). Therapeutic Anti-Tumor Efficacy of DC-Based Vaccines Targeting TME-Associated Antigens Is Improved When Combined with a Chemokine-Modulating Regimen and/or Anti-PD-L1. Vaccines.

[163] Qian C., Liu C., Liu W., Zhou R., Zhao L. (2023). Targeting vascular normalization: A promising strategy to improve immune-vascular crosstalk in cancer immunotherapy. Front. Immunol..

[164] Gao R.L., Song J., Sun L., Wu Z.X., Yi X.F., Zhang S.L., Huang L.T., Ma J.T., Han C.B. (2022). Efficacy and safety of combined immunotherapy and antiangiogenesis with or without chemotherapy for advanced non-small-cell lung cancer: A systematic review and pooled analysis from 23 prospective studies. Front. Pharmacol..

[165] Agarwal N., McGregor B., Maughan B.L., Dorff T.B., Kelly W., Fang B., McKay R.R., Singh P., Pagliaro L., Dreicer R. (2022). Cabozantinib in combination with atezolizumab in patients with metastatic castration-resistant prostate cancer: Results from an expansion cohort of a multicentre, open-label, phase 1b trial (COSMIC-021). Lancet Oncol..

[166] Wang H., Zhou F., Qin W., Yang Y., Li X., Liu R. (2025). Metabolic regulation of myeloid-derived suppressor cells in tumor immune microenvironment: Targets and therapeutic strategies. Theranostics.

[167] Zhu W., Chu Y., Gao P. (2025). Metabolic-immune nexus in tumor microenvironment: From mechanistic insights to therapeutic opportunities. Chin. Med. J..

[168] Osborne N., Sundseth R., Burks J., Cao H., Liu X., Kroemer A.H., Sutton L., Cato A., Smith J.P. (2019). Gastrin vaccine improves response to immune checkpoint antibody in murine pancreatic cancer by altering the tumor microenvironment. Cancer Immunol. Immunother..

[169] Padron L.J., Maurer D.M., O’Hara M.H., O’Reilly E.M., Wolff R.A., Wainberg Z.A., Ko A.H., Fisher G., Rahma O., Lyman J.P. (2022). Sotigalimab and/or nivolumab with chemotherapy in first-line metastatic pancreatic cancer: Clinical and immunologic analyses from the randomized phase 2 PRINCE trial. Nat. Med..

[170] Makgoba T.B., Kapp E., Egieyeh S., Joubert J. (2024). HDAC3 inhibitors: A patent review of their broad-spectrum applications as therapeutic agents. Expert Opin. Ther. Pat..

[171] Galluzzi L., Guilbaud E., Schmidt D., Kroemer G., Marincola F.M. (2024). Targeting immunogenic cell stress and death for cancer therapy. Nat. Rev. Drug Discov..

[172] Wang Y., Li Y., Yang Y., Swift M., Zhang Z., Wu S., Sun Y., Yang K. (2024). In situ vaccination caused by diverse irradiation-driven cell death programs. Theranostics.

[173] Golden E.B., Demaria S., Schiff P.B., Chachoua A., Formenti S.C. (2013). An abscopal response to radiation and ipilimumab in a patient with metastatic non-small cell lung cancer. Cancer Immunol. Res..

[174] Pant S., Wainberg Z.A., Weekes C.D., Furqan M., Kasi P.M., Devoe C.E., Leal A.D., Chung V., Basturk O., VanWyk H. (2024). Lymph-node-targeted, mKRAS-specific amphiphile vaccine in pancreatic and colorectal cancer: The phase 1 AMPLIFY-201 trial. Nat. Med..

[175] Wainberg Z.A., Weekes C.D., Furqan M., Kasi P.M., Devoe C.E., Leal A.D., Chung V., Perry J.R., Kheoh T., McNeil L.K. (2025). Lymph node-targeted, mKRAS-specific amphiphile vaccine in pancreatic and colorectal cancer: Phase 1 AMPLIFY-201 trial final results. Nat. Med..

[176] Williamson C.W., Sherer M.V., Zamarin D., Sharabi A.B., Dyer B.A., Mell L.K., Mayadev J.S. (2021). Immunotherapy and radiation therapy sequencing: State of the data on timing, efficacy, and safety. Cancer.

[177] Cao X.J., He X.Y., Yang Y., Zhu Q. (2026). Radiotherapy and immunotherapy for advanced cancers: A meta-analysis of dose, sequencing, and survival patterns. Transl. Oncol..

[178] Wang X., Maeng H.M., Lee J., Xie C. (2022). Therapeutic Implementation of Oncolytic Viruses for Cancer Immunotherapy: Review of Challenges and Current Clinical Trials. J. Biomed. Sci. Res..

[179] Stachura P., Stencel O., Lu Z., Borkhardt A., Pandyra A.A. (2023). Arenaviruses: Old viruses present new solutions for cancer therapy. Front. Immunol..

[180] Musher B.L., Rowinsky E.K., Smaglo B.G., Abidi W., Othman M., Patel K., Jawaid S., Jing J., Brisco A., Leen A.M. (2024). LOAd703, an oncolytic virus-based immunostimulatory gene therapy, combined with chemotherapy for unresectable or metastatic pancreatic cancer (LOKON001): Results from arm 1 of a non-randomised, single-centre, phase 1/2 study. Lancet Oncol..

[181] Lin C., Teng W., Tian Y., Li S., Xia N., Huang C. (2024). Immune landscape and response to oncolytic virus-based immunotherapy. Front. Med..

[182] Kollmann C.F., van Montfoort N., Cordelier P., Pol J., Olagnier D. (2025). Oncolytic virotherapy: Sparking durable anti-tumor immunity through microenvironment modulation. Semin. Immunol..

[183] de Bont D.A., Reugebrink M., van Montfoort N., Kemp V. (2026). Repurposing licensed viral vaccines as anti-cancer therapeutics: Turning cold tumors hot. Hum. Vaccines Immunother..

[184] Hasan N., Aftab M., Ikram S., Mustofa A.Z., Sriwidodo S., Darusman H.S., Amir M.N., Tockary T.A., Uchida S. (2026). Recent advances in lipid nanoparticles for cancer vaccine delivery: Challenges and future perspectives. Int. J. Pharm. X.

[185] Chheda D., Shete S., Tanisha T., Devrao Bahadure S., Sampathi S., Junnuthula V., Dyawanapelly S. (2024). Multifaceted therapeutic applications of biomimetic nanovaccines. Drug Discov. Today..

[186] Sheikhhossein H.H., Iommelli F., Di Pietro N., Curia M.C., Piattelli A., Palumbo R., Roviello G.N., De Rosa V. (2024). Exosome-like Systems: From Therapies to Vaccination for Cancer Treatment and Prevention-Exploring the State of the Art. Vaccines.

[187] Xu F., Yuan Y., Wang Y., Yin Q. (2023). Emerging peptide-based nanovaccines: From design synthesis to defense against cancer and infection. Biomed. Pharmacother..

[188] Gupta R., Shah K., Dewangan H.K. (2026). Nanovaccines in Cancer Immunotherapy: Lymph Node-Targeted Strategies and Mechanistic Insights. Curr. Pharm. Des..

[189] Wang Q., Wang Z., Sun X., Jiang Q., Sun B., He Z., Zhang S., Luo C., Sun J. (2022). Lymph node-targeting nanovaccines for cancer immunotherapy. J. Control Release.

[190] Dou L., Meng X., Yang H., Dong H. (2021). Advances in technology and applications of nanoimmunotherapy for cancer. Biomark. Res..

[191] Wang T., Wang Y., Liu T., Yu F., Liu L., Xiong H., Xu W., Fan X., Liu X., Jiang H. (2024). Potentiating Immunogenic Cell Death in Cold Tumor with Functional Living Materials of FeAu-Methylene Blue Composites. Adv. Healthc. Mater..

[192] Quiroga D., Lyerly H.K., Morse M.A. (2016). Deficient Mismatch Repair and the Role of Immunotherapy in Metastatic Colorectal Cancer. Curr. Treat. Options Oncol..

[193] Yuan Z., Liu Z., Zhou M., Wen H., Li B. (2026). Research progress on immunotherapeutics for triple-negative breast cancer from a single-cell perspective. Crit. Rev. Oncol. Hematol..

[194] Xia F., Yi Q., Xu Z., Zhou Z., Tang H., Zhang K., Yan Y. (2025). Microbial modulation as a game changer: Boosting immunotherapy efficacy in breast cancer. Semin. Cancer Biol..

[195] Takahashi H., Kojima D., Watanabe M. (2025). Therapeutic potential of trained immunity for malignant disease. Immunol. Med..

[196] Haist M., Stege H., Grabbe S., Bros M. (2021). The Functional Crosstalk between Myeloid-Derived Suppressor Cells and Regulatory T Cells within the Immunosuppressive Tumor Microenvironment. Cancers.

[197] Wang J., Zhang W., Zhang J., Zhao C., Zhang W., Fang M., Chen F., Wu Z., Xu X., Yu Z. (2026). Integrative Multi-Omics Analysis Uncovers Immunological Phenotypes Predictive of Combinatorial Immunotherapy Response in Gastric Cancer. Adv. Sci..

[198] Tiwari A., Oravecz T., Dillon L.A., Italiano A., Audoly L., Fridman W.H., Clifton G.T. (2023). Towards a consensus definition of immune exclusion in cancer. Front. Immunol..

[199] Le D.T., Diaz L.A., Kim T.W., Van Cutsem E., Geva R., Jager D., Hara H., Burge M., O’Neil B.H., Kavan P. (2023). Pembrolizumab for previously treated, microsatellite instability-high/mismatch repair-deficient advanced colorectal cancer: Final analysis of KEYNOTE-164. Eur. J. Cancer..

[200] Simnica D., Kobold S. (2022). Neoantigen T-Cell Receptor Gene Therapy in Pancreatic Cancer. N. Engl. J. Med..

[201] Sun S., Liu L., Zhang J., Sun L., Shu W., Yang Z., Yao H., Zhang Z. (2025). The role of neoantigens and tumor mutational burden in cancer immunotherapy: Advances, mechanisms, and perspectives. J. Hematol. Oncol..

[202] Yuan D.Y., McKeague M.L., Raghu V.K., Schoen R.E., Finn O.J., Benos P.V. (2025). Immune cell transcriptional profiles from pre-vaccination peripheral blood predict immune response to preventative MUC1 cancer vaccine. Eur. J. Cancer..

[203] Kazerani M., Mahov S., Foley J., Merin N.M., Choi S.Y., Lownik J., Xu A.M., Milshteyn L., Villamejor A.L., Nadri M. (2025). Pre-vaccine immune profiling in cancer patients identifies correlates of COVID-19 vaccine responses. Hum. Vaccines Immunother..

[204] Tazzari M., Carloni S., Bulgarelli J., Pignatta S., Bocchini M., Piccinini C., Angeli D., Tebaldi M., Azzali I., Tumedei M.M. (2026). Vaccine-expanded plasmablast-like B cells are associated with response to dendritic cell therapy in metastatic melanoma. J. Exp. Clin. Cancer Res..

[205] Ni Z., Ye D. (2026). The impact of gut microbiota modulation on responses to immune checkpoint inhibitors in cancer. Acta Microbiol. Immunol. Hung..

[206] Li S., Che C., Zhou Y., Fan D., Bai X., Lu Y., Zhao X. (2026). Gut microecology empowers cancer immunotherapy: Commensal microbiota-mediated mechanisms and translational prospects of PD-1/PD-L1 therapy. Cancer Biol. Med..

[207] Hilf N., Kuttruff-Coqui S., Frenzel K., Bukur V., Stevanovic S., Gouttefangeas C., Platten M., Tabatabai G., Dutoit V., van der Burg S.H. (2019). Actively personalized vaccination trial for newly diagnosed glioblastoma. Nature.

[208] Ascierto P.A., Brugarolas J., Buonaguro L., Butterfield L.H., Carbone D., Daniele B., Ferris R., Fox B.A., Galon J., Gridelli C. (2018). Perspectives in immunotherapy: Meeting report from the Immunotherapy Bridge (29–30 November, 2017, Naples, Italy). J. Immunother. Cancer..

[209] Routy B., Lenehan J.G., Miller W.H., Jamal R., Messaoudene M., Daisley B.A., Hes C., Al K.F., Martinez-Gili L., Punčochář M. (2023). Fecal microbiota transplantation plus anti-PD-1 immunotherapy in advanced melanoma: A phase I trial. Nat. Med..

[210] Vuscan P., Kischkel B., Joosten L.A.B., Netea M.G. (2025). Microbial-induced trained immunity for cancer immunotherapy. Pharmacol. Rev..

[211] Hsieh C.H., Chuang P.C., Liu Y.W. (2025). Beyond Adaptive Immunity: Trained Innate Immune Responses as a Novel Frontier in Hepatocellular Carcinoma Therapy. Cancers.

[212] Tao W., Sun Q., Xu B., Wang R. (2025). Towards the Prediction of Responses to Cancer Immunotherapy: A Multi-Omics Review. Life.

[213] Phung L.H., Nejo T., Okada H. (2024). Lessons from Post-Immunotherapy Tumor Tissues in Clinical Trials: How Can We Fuel the Tumor Microenvironment in Gliomas?. Vaccines.

[214] Aung T.N., Monkman J., Warrell J., Vathiotis I., Bates K.M., Gavrielatou N., Trontzas I.P., Tan C.W., Fernandez A.I., Moutafi M. (2025). Spatial signatures for predicting immunotherapy outcomes using multi-omics in non-small cell lung cancer. Nat. Genet..

[215] Sethna Z., Guasp P., Reiche C., Milighetti M., Ceglia N., Patterson E., Lihm J., Payne G., Lyudovyk O., Rojas L.A. (2025). RNA neoantigen vaccines prime long-lived CD8(+) T cells in pancreatic cancer. Nature.

[216] Qi C., Zhang P., Liu C., Zhang J., Zhou J., Yuan J., Liu D., Zhang M., Gong J., Wang X. (2024). Safety and Efficacy of CT041 in Patients with Refractory Metastatic Pancreatic Cancer: A Pooled Analysis of Two Early-Phase Trials. J. Clin. Oncol..

[217] Shitara K., Janjigian Y.Y., Ajani J., Moehler M., Yao J., Wang X., Chhibber A., Pandya D., Shen L., Garrido M. (2025). Nivolumab plus chemotherapy or ipilimumab in gastroesophageal cancer: Exploratory biomarker analyses of a randomized phase 3 trial. Nat. Med..

[218] Antoniotti C., Rossini D., Pietrantonio F., Salvatore L., Lonardi S., Tamberi S., Marmorino F., Moretto R., Prisciandaro M., Tamburini E. (2024). Upfront Fluorouracil, Leucovorin, Oxaliplatin, and Irinotecan Plus Bevacizumab with or Without Atezolizumab for Patients with Metastatic Colorectal Cancer: Updated and Overall Survival Results of the ATEZOTRIBE Study. J. Clin. Oncol..

[219] Gao C., Duan X., Peng L., Zhao Y., Li P., Mou K. (2025). Melanoma vaccines: Current R&D landscape, translational hurdles, and future outlook-a perspective drawn from 442 clinical trials. Front. Immunol..

[220] Haanen J.B., Schumacher T.N.M., Kagan J.C., Fritsch E.F., Wu C.J. (2026). Clinical development of cancer vaccines. Nat. Med..

[221] Zhang M., Liu C., Tu J., Tang M., Ashrafizadeh M., Nabavi N., Sethi G., Zhao P., Liu S. (2025). Advances in cancer immunotherapy: Historical perspectives, current developments, and future directions. Mol. Cancer..

[222] Liu Z., Lv J., Dang Q., Liu L., Weng S., Wang L., Zhou Z., Kong Y., Li H., Han Y. (2022). Engineering neoantigen vaccines to improve cancer personalized immunotherapy. Int. J. Biol. Sci..

[223] Xie N., Shen G., Gao W., Huang Z., Huang C., Fu L. (2023). Neoantigens: Promising targets for cancer therapy. Signal Transduct. Target. Ther..

[224] Hotchkiss K.M., Batich K.A., Mohan A., Rahman R., Piantadosi S., Khasraw M. (2023). Dendritic cell vaccine trials in gliomas: Untangling the lines. Neuro Oncol..

[225] Lu L., Lu X., Luo W. (2025). Personalized Cancer Vaccines: Current Advances and Emerging Horizons. Vaccines.

[226] Liao T., Chen X., Qiu F., Zhang X., Wu F., Zhao Z., Xu M., Chen M., Shen J.W., Shen Q. (2025). Regulation of cancer-associated fibroblasts for enhanced cancer immunotherapy using advanced functional nanomedicines: An updated review. J. Nanobiotechnology..

[227] Altalbawy F.M.A., Hussein U.A., Ballal S., Chahar M., Darwish N.S.A., Saini S., Prasad G.V.S., Rizaev J., Al-Anbari H.H.A., Saud M.J. (2025). Stromal reprogramming in solid tumors by nanoparticles: A review. Bioorg. Chem..

[228] Tong X., Tang R., Xu J., Wang W., Du Q., Shi S., Yu X. (2025). Cancer type-specific adverse events of immune checkpoint inhibitors: A systematic review and meta-analysis. Heliyon.

[229] Wolchok J.D., Kluger H., Callahan M.K., Postow M.A., Rizvi N.A., Lesokhin A.M., Segal N.H., Ariyan C.E., Gordon R.A., Reed K. (2013). Nivolumab plus ipilimumab in advanced melanoma. N. Engl. J. Med..

[230] Valsecchi M.E. (2015). Combined Nivolumab and Ipilimumab or Monotherapy in Untreated Melanoma. N. Engl. J. Med..

[231] Singh S., Banerjee M., Kushwah A.S. (2026). Personalized neoantigen cancer vaccines: Why clinical benefit remains inconsistent. Crit. Rev. Oncol. Hematol..

[232] Xu S., Yang H., Minev B., Ma W. (2026). Biomimetic and personalized nanovaccines in cancer immunotherapy: Design innovations, translational challenges, and future directions. J. Adv. Res..

[233] Wawrzak-Pienkowska K., Pienkowski T., Tankiewicz-Kwedlo A., Ciborowski M., Kurek K., Pawlak D. (2025). Differences in treatment outcome between translational platforms in developing therapies for gastrointestinal cancers. Eur. J. Pharmacol..

[234] Vaskovich-Koubi D., Green Buzhor M., Krinsky A., Roth Y., Salomon K., Kleiner R., Sevostianov R., Hasin O., Khoury R., Satchi-Fainaro R. (2025). Patient-Derived 3D-Bioprinted Models of Pancreatic Cancer: Toward Personalized Therapy and Overcoming Tumor Microenvironment Challenges. Adv. Drug Deliv. Rev..

[235] Hou Y., Chen C., Chen X. (2026). Advancing precision immuno-oncology in melanoma: The synergistic convergence of personalized neoantigen vaccines and multi-omics biomarker profiling. Front. Immunol..

[236] Gao Y., Gao Y., Wu S., Li D., Zhou C., Meng F., Dong K., Zhao X., Li P., Liang A. (2025). Weakly supervised peptide-TCR binding prediction facilitates neoantigen identification. Cell Syst..

[237] Passardi A., Sullo F.G., Bittoni A., Matteucci L., De Rosa F., Bulgarelli J., Tazzari M., Petrini M., Scarpi E., Testoni S. (2025). CombiCoR-Vax trial: Study protocol for a phase II, single-arm, multicenter trial of sequential pembrolizumab plus dendritic cell vaccine followed by trifluridine/tipiracil and bevacizumab in refractory microsatellite-stable metastatic colorectal cancer. BMC Cancer.

[238] Li X., Jabbarzadeh Kaboli P., Roozitalab G., Salahi S., Qian H., Zhang X., Chen K., Imani S. (2026). Next-generation neoantigen mRNA vaccines: Immuno-engineering strategies for precision cancer immunotherapy. Cell. Oncol..

[239] Magoola M., Niazi S.K. (2025). Current Progress and Future Perspectives of RNA-Based Cancer Vaccines: A 2025 Update. Cancers.

[240] Sundaram M.E. (2026). Vaccine safety for individuals receiving immune checkpoint inhibitor therapy: A narrative review of current literature and recommendations for future research. Hum. Vaccines Immunother..

[241] Lu M., Liu Y., Xu L., Gao Y., Liu P., Liu Z., Tan X., Li W., Lin Y., Chen L. (2025). OmniNeo: A multi-omics pipeline incorporating proteomics and AI selection for neoantigen optimization in tumor immunotherapy. Front. Immunol..

[242] Vasudevan K., T D., Hebbar S.R., Selvam P.K., Rambabu M., Anbarasu K., Rohini K. (2025). Multi-omics and AI-driven immune subtyping to optimize neoantigen-based vaccines for colorectal cancer. Sci. Rep..

[243] Srivastava R. (2026). AI-powered mapping of tumor immunity for optimized mRNA vaccine engineering. Front. Oncol..

[244] Lei D., Wang W., Zhao J., Zhou Y., Chen Y., Dai J., Qiu Y., Qi H., Li C., Liang B. (2025). An injectable gambogic acid loaded nanocomposite hydrogel enhances antitumor effect by reshaping immunosuppressive tumor microenvironment. Mater. Today Bio..

[245] Yan Y., Lu J., Luo H., Wang Z., Xu K., Wang L., Wang Q. (2025). Decoding immune low-response states in ovarian cancer: Insights from single-cell and spatial transcriptomics for precision immunotherapy. Front. Immunol..

[246] Kalogirou C., Krebs M., Kunz A.S., Hahn O., Kubler H., Schwinger M., Hartmann A., Schmieder A., Eckstein M. (2025). Spatial transcriptomic profiling of metastatic renal cell carcinoma identifies chemokine-driven macrophage and CD8+ T-cell interactions predictive of immunotherapy response. J. Immunother. Cancer.

[247] Kim S.H., Choi W., Kim Y., Kim H., Jeong G.W., Park H., Youn I., Park Y., Han S., Kim Y.M. (2026). Remote-Controllable Immune-Priming Spot Formation Via Nanocomplexed Hydrogel-Assisted Histotripsy: Immunoasis. Adv. Mater..

[248] Bird L. (2025). BCG plus beta-glucan trains neutrophils to beat bladder cancer. Nat. Rev. Immunol..

[249] Swamy K. (2022). Vascular normalization and immunotherapy: Spawning a virtuous cycle. Front. Oncol..

